# WTAP facilitates progression of hepatocellular carcinoma via m6A-HuR-dependent epigenetic silencing of ETS1

**DOI:** 10.1186/s12943-019-1053-8

**Published:** 2019-08-22

**Authors:** Yunhao Chen, Chuanhui Peng, Junru Chen, Diyu Chen, Beng Yang, Bin He, Wendi Hu, Yanpeng Zhang, Hua Liu, Longfei Dai, Haiyang Xie, Lin Zhou, Jian Wu, Shusen Zheng

**Affiliations:** 1 Division of Hepatobiliary and Pancreatic Surgery, Department of Surgery, First Affiliated Hospital, School of Medicine, Zhejiang University, Hangzhou, China; 2 Key Laboratory of Combined Multi-Organ Transplantation, Ministry of Public Health, Hangzhou, China; 3 Key Laboratory of Organ Transplantation, Hangzhou, Zhejiang Province China; 4 Key Laboratory of the Diagnosis and Treatment of Organ Transplantation, CAMS, Hangzhou, China

**Keywords:** N6-methyladenosine (m6A), Wilms tumor 1-associated protein (WTAP), Hepatocellular carcinoma (HCC), ETS1

## Abstract

**Background:**

N6-methyladenosine (m6A) methylation, a well-known modification with new epigenetic functions, has been reported to participate in the tumorigenesis of hepatocellular carcinoma (HCC), providing novel insights into the molecular pathogenesis of this disease. However, as the key component of m6A methylation, Wilms tumor 1-associated protein (WTAP) has not been well studied in HCC. Here we investigated the biological role and underlying mechanism of WTAP in liver cancer.

**Methods:**

We determined the expression of WTAP and its correlation with clinicopathological features using tissue microarrays and the Cancer Genome Atlas (TCGA) dataset. And we clarified the effects of WTAP on HCC cells using cell proliferation assay, colony formation, Edu assay and subcutaneous xenograft experiments. We then applied RNA sequencing combined with gene expression omnibus (GEO) data to screen candidate targets of WTAP. Finally, we investigated the regulatory mechanism of WTAP in HCC by m6A dot blot assay, methylated RNA immunoprecipitation (MeRIP) assay, dual luciferase reporter assay, RNA immunoprecipitation (RIP) assay and Chromatin immunoprecipitation (ChIP) assay.

**Results:**

We demonstrated that WTAP was highly expressed in HCC which indicated the poor prognosis, and that WTAP expression served as an independent predictor of HCC survival. Functionally, WTAP promoted the proliferation capability and tumor growth of HCC cells in vitro and in vivo. Furthermore, ETS proto-oncogene 1 (ETS1) was identified as the downstream effector of WTAP. The m6A modification regulated by WTAP led to post-transcriptional suppression of ETS1, with the implication of Hu-Antigen R (HuR) as an RNA stabilizer. Then ETS1 was found to inhibit the progression of HCC and could rescue the phenotype induced by WTAP deficiency. Moreover, WTAP modulated the G2/M phase of HCC cells through a p21/p27-dependent pattern mediated by ETS1.

**Conclusion:**

We have identified that WTAP is significantly up-regulated in HCC and promotes liver cancer development. WTAP-guided m6A modification contributes to the progression of HCC via the HuR-ETS1-p21/p27 axis. Our study is the first to report that WTAP-mediated m6A methylation has a crucial role in HCC oncogenesis, and highlights WTAP as a potential therapeutic target of HCC treatment.

**Electronic supplementary material:**

The online version of this article (10.1186/s12943-019-1053-8) contains supplementary material, which is available to authorized users.

## Background

Hepatocellular carcinoma (HCC), one of the most prevalent malignancies in adults, ranks the second among leading causes of tumor-related death worldwide [1]. Owing to the high rate of recurrence and metastasis, HCC patients are commonly diagnosed with a poor prognosis, especially at advanced stages [2]. Although treatments for HCC have witnessed immense progress over the recent decades, ranging from interventional therapy, curative resection, or liver transplantation to targeted therapy or immunotherapy, outcomes of HCC are still undesirable [3]. Thus, it is imperative to further elucidate the molecular pathogenesis of HCC in order to develop novel therapeutic strategies and reduce the mortality of this malignancy.

Abundant evidence has demonstrated that epigenetic dysregulation substantially contributes to the development of HCC [3], while major investigations are focused at the transcriptional level [4]. Nowadays, a sort of reversible post-transcriptional modification located in RNA (called RNA methylation) has drawn increasing attention. Among which, N6-methyadenosine (m6A) modification is the most predominant type of messenger RNA (mRNA) methylation in mammals [5]. Approximately 0.1–0.4% of adenosines in total RNA are modified by m6A methylation [6]. The consensus motif of m6A is identified as RRACH ([R: G/A/U] [R: G/A] AC [H: U/A/C]) [7], and m6A sites are mainly enriched in 3′ untranslated regions (3′ UTRs) and near stop codons according to the high-throughput m6A profiling [8]. Effects of m6A are accomplished by the dynamic interaction of “writers” (methyltransferases), “erasers” (demethylases) and “readers” (effector proteins). The classical complex of writers consists of methyltransferase-like 3 (METTL3), methyltransferase-like 14 (METTL14) and Wilms tumor 1-associated protein (WTAP) [9]. METTL3 is the crucial component of methyltransferases as an S-adenosylmethionine (SAM)-binding subunit, while METTL14 serves as an RNA-binding scaffold for substrate recognition [10]. And WTAP is indispensable for stabilization of METTL3 and METTL14, and their localization into nuclear speckles [9]. Recently, more components of writers have been recognized such as METTL16 [11], KIAA1429 [12], RBM15, RBM15B [13], Zc3h13 and HAKAI [14, 15]. On the other side, AlkB homolog 5 (ALKBH5) and fat mass and obesity-associated (FTO) act as the erasers with m6A demethylation activity [16, 17]. The readers are comprised of YTH family which embraces domain of YT521-B homology (YTHDF1–3, YTHDC1–2) [18–21], heterogeneous nuclear ribonucleoprotein (HNRP) family (HNRNPA2B1, HNRNPC) [22, 23], and insulin-like growth factor 2 mRNA-binding proteins (IGF2BP1/2/3) [24]. They are mainly involved in m6A-containing mRNA stability or metabolism and protein translation efficiency [18, 19].

m6A modification is implicated in diverse biological processes including tumorigenesis. Several members (e.g. METTL3, METTL14, FTO, ALKBH5 and YTHDF2) actively participate in human cancers such as acute myeloid leukaemia (AML) [25], glioblastoma [26], breast cancer [27] and endometrial cancer [28]. Multiple functions, ranging from tumor initiation, development and metastasis to cancer stem cell pluripotency, are mediated by m6A methylation. Particularly for HCC, METTL14 is primarily reported to be a tumor suppressor through manipulating the processing of m6A-modified pri-miR126 with the aid of DGCR8 [29]. In addition, METTL3 reinforces the progression of HCC by intensifying the m6A-modification of SOCS2 via an YTHDF2-dependent manner [30]. Nevertheless, the role of WTAP, another essential player in the methyltransferase complex, has been poorly understood in HCC.

WTAP is a conserved nuclear protein as the partner of Wilms’ tumor 1 (WT1) [31]. Removal of WTAP has been proved to be embryonic lethal [32], which indicates its vital biological character in the development of vertebrates. It has been reported that WTAP is involved in substantial cellular processes, such as alternative splicing [33], X-chromosome inactivation [34] and cell cycle regulation [32]. Moreover, accumulating evidence suggests that WTAP contributes to aggressive features of numerous cancers. For instance, overexpression of WTAP facilitates renal cell carcinoma by stabilizing CDK2 transcript [35], and promotes the invasiveness of glioblastoma through enhancing the activity of EGFR [36]. WTAP is also an oncogene in both AML and diffuse large B-cell lymphoma which are associated with heat shock protein 90 [37, 38]. Regarding cholangiocarcinoma, WTAP strengthens its aggressiveness by stimulating the expression of metastasis-related markers MMP7 and MMP28 [39]. However, the biological role of WTAP relevant to m6A modification in HCC has not yet been illustrated.

In the current study, the up-regulation of WTAP in HCC tissues compared with normal adjacent tissues was first identified, which was associated with tumor progression and poorer survival in HCC patients. We further demonstrated WTAP to be an oncogenic protein that enhanced the proliferation and invasiveness of HCC in vitro and in vivo. Mechanistically, WTAP-mediated m6A modification led to the epigenetic silencing of ETS proto-oncogene 1 (ETS1), which was subsequently validated as a tumor suppressor in HCC, with the involvement of Hu-Antigen R (HuR) to stabilize ETS1 mRNA. Further investigations revealed that WTAP affected the cell cycle dependent on p21 and p27, which were the downstream of ETS1 simultaneously.

## Materials and methods

### Patients and specimens

Samples of tumor tissues and adjacent tissues were gathered from 69 HCC patients who had received curative surgery from 2016 to 2017 at First Affiliated Hospital of Zhejiang University. Frozen tissues were subjected to mRNA or protein extraction for reverse transcription quantitative real-time PCR (RT-qPCR) or western blotting analysis, and a cohort including 32 pairs of paraffin-embedded tissues (without follow-up data) (named as cohort1) was applied for immunohistochemistry (IHC) analysis. Another cohort was based on tissue microarrays (TMA) which was purchased from Shanghai Outdo Biotech (HLivH180Su14) containing 90 pairs of HCC tissues and matching normal tissues with complete clinicopathological information and follow-up data (listed detailedly in Table 1). Written informed consents were obtained from each enrolled subject according to the guidelines of the Declaration of Helsinki. And this study was approved by Institutional Ethics Committee in the hospital.

**Table 1:** Clinical characteristics of 90 HCC patients depending on WTAP expression levels

Feture	WTAP-high	WTAP-low	n	*Χ* ^*2*^	*P*
All cases	45	45			
Differentiation				1.113	0.291
Low	19	24	43		
High	26	21	47		
AJCC stage				0.194	0.66
Stage I	30	28	58		
Stage II & III	15	17	32		
Tumor size				0.809	0.362
≤ 5 cm	29	33	62		
>5 cm	16	12	28		
Tumor encapsulation				3.607	0.045*
Absent	28	19	47		
Present	17	26	43		
Microvascular infiltration				0.104	0.747
Absent	30	28	58		
Present	10	11	21		
HbsAg				0.338	0.561
Negative	8	10	18		
Positive	37	34	71		
HbcAb				0.09	0.765
Negative	4	3	7		
Positive	41	39	80		
AFP				0.647	0.421
≤ 400 μg/L	27	30	57		
>400 μg/L	18	14	32		
Recurrence				3.629	0.045*
No	16	25	41		
Yes	29	20	49		

*Χ*^*2*^ Test was used to test the association between categorical variables

*Statistically significant

### Cell culture

The human HCC cell lines Huh7, PLC/PRF/5, Hep3B, HCCLM3, MHCC97H, SMCC7721 and an immortalized hepatocyte cell line QSG7701 were purchased from Shanghai Institutes of Biological Sciences, Chinese Academy of Sciences (Shanghai, China). All cells were routinely cultured with Minimum Essential Media (MEM, Gibco) supplemented with 10% fetal bovine serum (FBS, Gibco) and incubated in 5% CO2, 37 °C incubator (Thermo Scientific, USA) with a humidified atmosphere.

### RNA extraction and RT-qPCR

Total RNA was isolated using ESscience RNA-Quick Purification Kit (YiShan Biotech, Shanghai, China), followed by cDNA synthesis with HiScript II Q RT SuperMix for qPCR (Vazyme Biotech, Nanjing, China). Expression of RNA was measured by SYBR Green (Vazyme Biotech, Nanjing, China) operated on the Bio-Rad QX100 Droplet Digital PCR system (USA), and relative RNA amount was calculated by 2^ΔΔCt^ method with the normalization to GAPDH. All premiers were derived from Tsingke Biological Technology (Beijng, China) and summarized in Additional file 1: Table S1.

### Western blotting

Total protein was extracted from tissues or cells using pre-cooled RIPA buffer (Beyotime, Shanghai, China) containing protease and phosphatase inhibitors (Thermo Scientific, USA). Quantification of protein was conducted with Bicinchoninic Acid Protein Assay Kit (Thermo Scientific, USA). An equal amount of protein samples was separated by 4–12% SDS-PAGE (GenScript, Nanjing, China) and then transferred to 0.45 μm PVDF membranes (Millipore, USA). After being blocked by 5% non-fat milk in TBST for 1 h, membranes were incubated with corresponding primary antibodies at 4 °C overnight. Washed by TBST for three times, they would be incubated with HRP-conjugated secondary antibodies for 1 h at room temperature. The immunoblots were detected with an imaging system (Bio-Rad, USA) using enhanced chemiluminescence detection kit (Servicebio, Wuhan, China). GAPDH and β-actin were selected to be the loading controls. All the antibodies employed in this study were listed in Additional file 2: Table S2.

### Immunohistochemistry

The TMA cohort was utilized to construct the connection between WTAP and prognosis of HCC patients, and the paraffin embedded specimens (cohort1) were applied to certify the relationship of WTAP and ETS1. They were subjected to IHC staining using two-step method of Dako Envision™ Detection System (DakoCytomation, Glostrup, Denmark). IHC score was computed via multiplying staining intensity grade (grade of 0, 1, 2 or 3 implied negative, weak-positive, moderate-positive or strong-positive, respectively) by positive rate score (score of 0, 1, 2, 3 or 4 represented positive areas of 0–5%, 6–25%, 26–50%, 51–75% or 76–100%, respectively). The score was assessed by two proficient pathologists independently.

### RNA interference

Small interfering RNAs (siRNA) directed against WTAP, ETS1, HuR, YTHDF2, ETS2 and negative control RNAs (siNC) were synthesized by GenePharma Company (Shanghai, China). Transient transfection was performed by jetPRIME Polyplus kit (France) in accordance with the standard protocol. Cells were harvested after 48 h for qRT-PCR examination or after 72 h for immunoblot analysis and functional study. All the siRNA sequences were generalized in Additional file 3: Table S3.

### Construction of stable knockdown and overexpressed cells

Lentiviruses expressing shWTAP (#1, #3) or shNC, Flag-WTAP (WTAP-OE) or empty vector (WTAP-NC), and Flag-ETS1 (ETS1-OE) or empty vector (ETS1-NC) were purchased from Genecopoeia (USA). Huh7 and PLC/PRF/5 cells were chosen to establish stable WTAP knockdown models, while SMMC-7721 and HCCLM3 cells were used for stable WTAP overexpression experiments. 10^5^ cells were planted into a well and transfected with indicated lentivirus according to the instructions. Infected cells were selected using 3 μg/ml puromycin (MCE, USA) for 1 week or more, with the transfection efficiency determined by RT-qPCR and WB analysis. All the targeted sequences were listed in Additional file 3: Table S3.

### Cell proliferation assay and colony formation

Cell proliferation ability was measured by Cell Counting Kit-8 (CCK-8, Dojindo Laboratories, Kumamoto, Japan). 2 × 10^3^ cells were seeded into a 96-well plate per well with six duplications, followed by incubation for 2 h at 37 °C. Absorbance was detected at 450 nm daily for 4 consecutive days. For colony formation assay, 1.5× 10^3^ treated cells were coated into 6-well plates with three repetitions. After 14-day incubation, these plates were washed with phosphate buffered saline (PBS) twice, fixed by methanol for 10 min and stained with 0.1% crystal violet solution within 10 min for further analysis.

### Cell cycle analysis

The treated cells were collected and fixed with chilled 75% ethanol at − 20 °C overnight or longer. After ethanol discarded, cells were washed twice with PBS and resuspended with 250 μl DNA staining solution (Multiscience, China) at room temperature for 30 min. Cell cycle analysis was performed on the flow cytometry (FACS LSRII, BD Bioscience, USA).

### Edu assay

A 5-ethynyl-20-deoxyuridine (EdU) assay kit (Ribobio, Guangzhou, China) was adopted to inquire the cell proliferation ability. Cells were seeded into confocal plates with a density of 10 × 10^5^ cells each well. And they were incubated with 50 μM EdU buffer at 37 °C for 2 h, fixed with 4% formaldehyde for 0.5 h and permeabilized with 0.1% Triton X-100 for 20 min. EdU solution was added into culture followed by the staining of nuclei with Hoechst. Then the results were visualized by a fluorescence microscope.

### Migration and invasion assays

Migration or invasion assays were performed using 24-well plates inserted by 8-μm pore size transwell filter insert (Corning, NY, USA) with or without pre-coated diluted Matrigel (1,10) (Becton Dickinson, San Jose, CA, US). 5 × 10^4^ HCC cells with Serum-free medium were placed into the upper chamber, and medium containing 10% FBS was added into the bottom chamber subsequently. After incubation in 37 °C for 24 h (migration) or 48 h (invasion), cells on the underside of membrane were immobilized and stained with crystal violet (Sangon Biotech, China). Then penetrated cells were counted in five random fields under the microscope.

### Subcutaneous xenograft experiments

All animal experiments were approved by the Ethics Committee for Laboratory Animals of the First Affiliated Hospital of Zhejiang University. Four-week old Balb/c male nude mice were purchased from Shanghai Experimental Animal Center of Chinese Academic of Sciences (Shanghai, China). 3 × 10^6^ treated HCC cells resuspended in 100 μl PBS were subcutaneously injected to the left flank of the mice, which were randomly divided into several groups. Tumor sizes were measured every 3 to 5 days. At the end of feeding (3 weeks or more), mice were sacrificed with the tumors removed for histology analysis. The tumor weight was recorded and the volume was estimated with the formula: 1/2*(length×width^2^). And extreme values (maximum and minimum) were eliminated.

### RNA sequencing

Total RNA was extracted with ESscience RNA-Quick Purification Kit (YiShan Biotech, Shanghai, China). Then the library construction and RNA-sequencing (RNA-seq) were performed at Shanghai Sinomics Corporation (Shanghai, China) with Illumina NovaSeq 6000 (Illumina, USA), followed by the computational analysis they provided. The criteria for differential genes was set up with *P* value < 0.01 and fold change > 1.5 or < 0.5.

### RNA total m6A quantification

Total RNA was extracted by ESscience RNA-Quick Purification Kit (YiShan Biotech, China), followed by the purification of polyadenylated mRNA using GenElute™ mRNA Miniprep Kit (Sigma-Aldrich, Germany) according to manufacturer’s protocol. Then EpiQuik™ m6A RNA Methylation Quantification Kit (Colorimetric) (Epigentek, USA) was utilized to determine the total level of m6A in treated cells. Briefly, 200 ng RNA was added to each well with the capture antibody and detection antibody mixed in subsequently. After several incubations, the m6A content was quantified colorimetrically at the wavelength of 450 nm and calculated based upon the standard curve.

### m6A dot blot assay

Total RNA or poly(A) + mRNA was isolated as described above. mRNA samples dissolved in 3 times volume of RNA incubation buffer were denatured at 65 °C within 5 min. Then the samples, divided into subgroups of 400 ng, 200 ng and 100 ng, were loaded to an Amersham Hybond-N+ membrane (GE Healthcare, USA) installed in a Bio-Dot Apparatus (Bio-Rad, USA) with the mixture of ice-cold 20*SSC buffer (Sigma-Aldrich, Germany). The membrane was UV crosslinked for 5 min and washed with PBST. Whereafter, it was stained with 0.02% Methylene blue (Sangon Biotech, China), followed by the scanning to indicate the total content of input RNA. After being blocked with 5% non-fat milk, the membrane was incubated with specific m6A antibody (1:1000, Millipore) overnight at 4 °C. Dot blots were hatched with HRP-conjugated anti-mouse immunoglobulin G (IgG) for 1 h before visualized by an imaging system (Bio-Rad, USA).

### RNA immunoprecipitation (RIP)

RIP assay was carried out in Huh7 and PLC/PRF/5 cells using Magna RIP Kit (17–700, Millipore, MA) following the manufacturer’s instructions. In brief, magnetic beads pre-coated with 5 mg normal antibodies against HuR (Santa Cruz) or mouse IgG (Millipore) were incubated with sufficient cell lysates (more than 2*10^7^ cells per sample) at 4 °C overnight. And the beads containing immunoprecipitated RNA-protein complex were treated with proteinase K to remove proteins. Then interested RNAs were purified by TRIzol methods (ThermoFisher Scientific) and detected by RT-qPCR with the normalization to input (fold change was calculated for comparison).

### Methylated RNA immunoprecipitation (MeRIP)

Cells with stable knockdown of WTAP were subjected to MeRIP assay using Magna MeRIP™ m6A Kit (17–10,499, Millipore, MA) in accordance with manufacturer’s recommendations. In short, 200 μg total RNA was isolated and randomly fragmented into 100 nucleotides or less, followed by the immunoprecipitation with 10μg m6A antibody (MABE1006, Millipore) or anti-mouse IgG which was linked to Magna ChIP Protein A/G Magnetic Beads. To determine the appropriate ratio between RNA and antibody, a dilution assay had been applied to optimize the MeRIP system and ensure the m6A antibody was not saturated. And one-tenth volume of fragmented RNA was saved as “10% input”. Elution of m6A-precipitated RNA was based on 6.7 mM N6-methyladenosine 5′-monophosphate sodium salt. And modification of m6A towards particular genes was determined by qPCR analysis with specific primers (All primers for MeRIP-qPCR were listed in Additional file 1: Table S1) Note: m6A sites of specific genes were predicted in RMBase v2.0 (http://rna.sysu.edu.cn/rmbase/) and SRAMP (http://www.cuilab.cn/sramp) (Additional file 5: Data S1). We focused on the potential m6A sites in 3′ UTR near the stop codon and designed primers to ensure that target sequence embodied all these sites with the limited length of 100 nt.

### Luciferase reporter assay

cDNAs containing partial CDS sequence near stop codon and full-length 3’UTR of ETS1 were cloned into pGL3-control vectors (Promega) which was comprised of firefly luciferase(F-luc). For mutant 1 or 2 reporter plasmids, 4 or 11 adenosine (A) in m6A motif were replaced by cytosine (C), respectively. The inserted sequences were listed in Additional file 6: Data S2. Pre-treated HCC cells were seeded into 24-well plate followed by co-transfection of 0.5μg of wild-type or mutated ETS1 reporter plasmids and 25 ng pRL-TK plasmids (renilla luciferase reporter vector) using jetPRIME Polyplus kit. After 24–36 h, cells were harvested to access the luciferase activity using Dual-Glo Luciferase system (Promega) with the normalization to pRL-TK. Each group was conducted in triplicate.

### RNA decay assay

To evaluate the RNA stability, RNA decay assay was conducted. HCC cells were cultured in four 6-well plates followed by the treatment of WTAP or HuR knockdown. Then Actinomycin D (MCE, HY-17559) was added into each well with a final concentration of 5 μg/mL. And cells were collected after 0, 1, 2, 4 h, respectively. Total RNA was isolated and subject to RT-qPCR subsequently to quantify the relative abundance of ETS1 mRNA (relative to 0 h).

### Chromatin immunoprecipitation (ChIP) assay

ChIP assay was carried out in Huh7 and MHCC97H cells with Magna ChIP™ A/G kit (17–10,085, Millipore, MA) according to manufacturer’s protocols. In brief, 10^7^ cells fixed with formaldehyde were collected and subject to 500ul lysis buffer. Then lysate was sonicated for 25 cycles of 6-s power-on and 30-s interval with intensity of 200 W. Afterwards, the supernatant was diluted and fully mixed with Protein A/G magnetic beads. Then 5μg of IgG, ETS1 or anti-RNA Polymerase II antibody was added in respectively, followed by incubation at 4 °C overnight. The next day, after washing, the mixture was incubated with elution buffer at 62 °C for 2 h and then at 95 °C for 10 min. Then DNA was purified from the elution and was subject to RT-qPCR. Specific primers for p21 and p27 was designed with the help of JASPAR (http://jaspar.genereg.net/) and PROMO (http://alggen.lsi.upc.es/).

### Treatment of methylation inhibitors

HCC cells were treated with 3-Deazaadenosine (DAA, B6121, APExBIO) in the concentration of 0, 100, 200uM, or with cyclolencine (A48105, Sigma-Aldrich) in the concentration of 0, 50, 100 mM for 24 or 48 h, followed by the RT-qPCR or western blotting analysis to examine the expression of ETS1 or ETS2.

### Co-immunoprecipitation

Co-Immunoprecipitation (Co-IP) assay was performed in Huh7 cells using Dynabeads™ Co-Immunoprecipitation Kit (14321D, ThermoFisher Scientific, USA). According to the manufacturer’s manuals, the protein extracted with IP Lysis Buffer was subject to beads premixed with antibodies of WTAP or IgG. The immunoprecipitated protein complex was separated from beads after several washes, followed by the identification for partners of WTAP by immunoblots.

### Immunofluorescence assay

Cells (4*10^4^) were cultured on confocal dishes, fixed with 4% formaldehyde for 20 min, and permeabilized with 0.1% Triton X-100 for 20 min. After blocking with 5% BSA (in TBST), cells were incubated with the primary antibodies against WTAP (proteintech, 1:100) and ETS1 (CST, 1:200) at 4 °C overnight. The secondary antibodies were then incubated with the cells at room temperature for 30 min, and DAPI was applied to stain the nuclei. Immunofluorescence (IF) images were obtained with confocal microscopy (Olympus, Japan).

### Nuclear and cytoplasmic protein extraction

The Nuclear and Cytoplasmic Protein Extraction Kit (Beyotime, Shanghai, China) was used to isolate the nuclear and cytoplasmic components of HCC cells. The detailed procedures were operated in accordance with the protocols of manufacturer. GAPDH or LaminB1 were served as loading controls for nuclear or cytosolic fraction, respectively.

### Statistical analysis

Statistical analyses were conducted with the SPSS 22.0 (SPSS, Inc., Chicago, IL, USA) and GraphPad Prism 7.0 software (GraphPad, Inc., La Jolla, CA, USA). Experiments were repeated at least for three times independently. Measured data were represented as the mean ± SD. One-way analysis of variance (ANOVA) or two-tail Student t test was applied to compare quantitative data, while the nonparametric χ2 test was used to analyze qualitative data. Cox proportional hazard regression model was employed for univariate or multivariate analysis to explore independent prognostic factors. The overall or disease-free survival was analyzed with Kaplan–Meier method, using the log-rank test to determine the difference. *P*-values for each analysis are marked on figures, and the level of statistical significance was defined to *P* < 0.05 (*P < 0.05; **P < 0.01; ***P < 0.001; ****P < 0.0001).

## Results

### Overexpression of WTAP correlated with poor prognosis of HCC

**Fig. 1:**
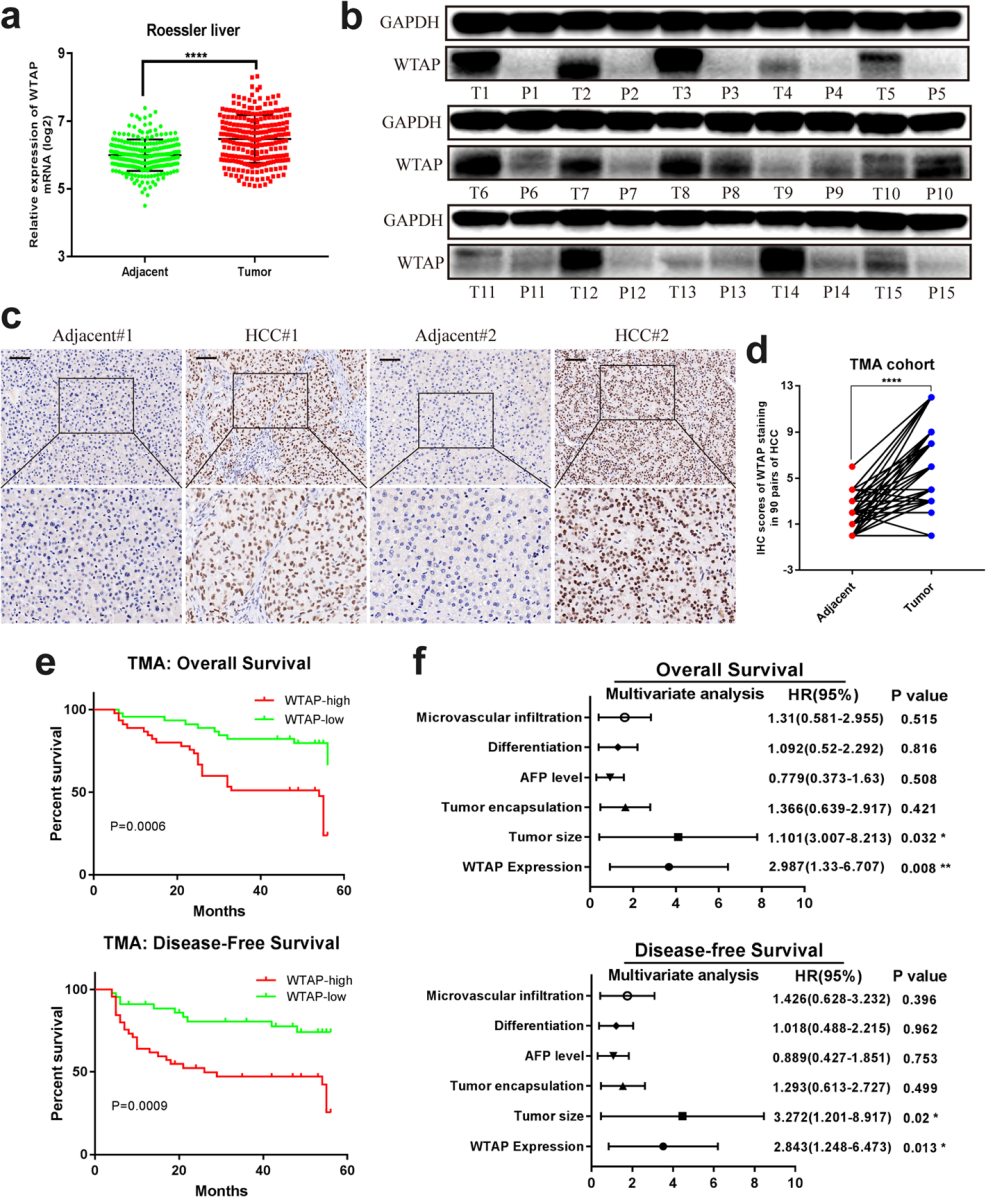
Up-regulated WTAP expression is associated with poor outcomes of HCC. **a** The expression of WTAP mRNA was determined based on GEO datasets (GSE14520); **b** The expression of WTAP protein was analyzed by western blotting in 15 pairs of HCC tissues; (T: tumor; P: peritumor); **c** Representative IHC images of WTAP staining in HCC tumor or adjacent tissues (scale bar, 100 μm; magnification, 200X and 400X); **d** IHC scores of 90 pairs of HCC tissues in the TMA cohort based on WTAP staining; **e** Kaplan-Meier analysis of overall survival and disease free survival of 90 HCC patients (data from TMA); **f** Forest plots based upon the outcomes of multivariate analysis of several factors associated with OS and RFS of HCC patients. Note: The factors that were closely associated with clinical outcomes of HCC were adopted into a COX regression model. Therefore, the restriction of statistical significance (*P* < 0.05) may be properly broadened

To clarify the role of WTAP, we first analyzed the mRNA expression of WTAP in human HCC samples from gene expression omnibus (GEO) datasets (Roessler liver) and the Cancer Genome Atlas (TCGA) data. The results showed conspicuously higher WTAP expression in tumor tissues (Fig. 1a and Additional file 7: Figure S1a). The WTAP protein level was also remarkably up-regulated in HCC tissues (Fig. 1b), which was further confirmed by IHC staining of TMA cohort (Fig. 1c, d). Moreover, we investigated the relationship between WTAP expression and clinicopathological features in 90 HCC patients. WTAP expression was significantly interrelated with tumor encapsulation and recurrence (Table 1). And Kaplan-Meier analysis revealed that patients with higher WTAP expression level were associated with poorer overall survival (OS) and disease-free survival (DFS) (Fig. 1e). Besides, the overexpression of WTAP was found to be an independent prognostic factor for OS (*p* = 0.008) and DFS (*p* = 0.013) in HCC patients (Fig. 1f). Taken together, we concluded that WTAP is up-regulated in HCC and is closely related to its poor prognosis.

### WTAP promoted tumor proliferation and tumorigenic ability in vitro and in vivo

**Fig. 2:**
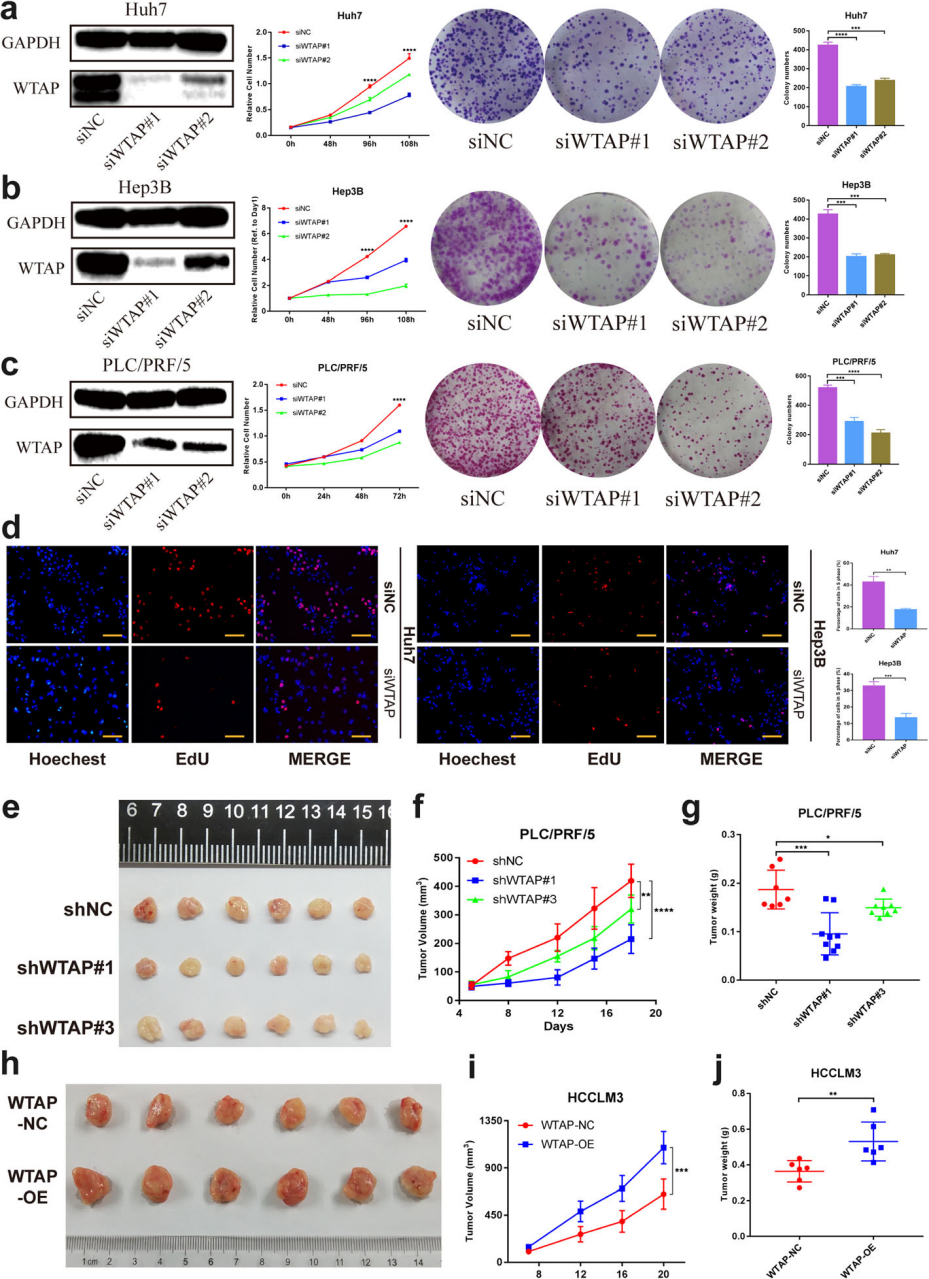
WTAP promotes tumor growth of HCC cells in vitro and in vivo. **a**, **b** and **c** Negative control or siRNA (si-WTAP #1, #2) was transfected into Huh7 (**a**), Hep3B (**b**) and PLC/PRF/5 (**c**) cells, respectively. The knockdown efficiency was tested and proliferation capacities were detected by CCK-8 and colony formation assay, with bar charts showing colony numbers; **d** EdU assay was applied to compare the cell proliferation ability in Huh7 and Hep3B (scale bar, 100 μm); **e**, **f** and **g** Tumor growth curve (**f**) of stable WTAP silenced PLC/PRF/5 cells (or negative control) in the xenograft mouse model was based on tumor size measurement. Moreover, tumor nodules (**e**) were collected and tumor weights (**g**) were recorded to present the growth difference within the influence of WTAP. **h**, **i** and **j** Tumor growth curve (**i**) of stable WTAP overexpressing HCCLM3 cells (or negative control) in the xenograft model was presented, followed by the collection of tumor nodules (**h**) and tumor weight records (**j**)

Expression of WTAP in HCC cell lines was examined before functional experiments (Additional file 7: Figure S1b, c). Knockdown and overexpression of WTAP were carried out in Huh7/Hep3B/PLC/PRF/5 and SMMC-7721/HCCLM3 cells, respectively. The CCK-8 and colony formation assays indicated that WTAP deficiency inhibited the proliferation ability in all three cell lines (Fig. 2a-c), and the results were reversed when WTAP was overexpressed (Additional file 7: Figure S1d, e). In addition, the EdU assay implied that cell growth was decreased with WTAP knockdown (Fig. 2d). Furthermore, cell migration and invasion were substantially impaired by silencing of WTAP (Additional file 7: Figure S1f, g).

To confirm the role of WTAP in vivo, tumor xenograft models were constructed by subcutaneously injecting HCC cells with either stable knockdown (shWTAP #1, #3) or overexpression of WTAP (WTAP-OE) into nude mice. We found that WTAP depletion repressed tumorigenesis (Fig. 2e) with prominently lower tumor volumes and weights compared with negative control group (Fig. 2f, g). And it also contributed to the down-regulated level of Ki67 in xenograft tumor tissues (Additional file 8: Figure S2a) Meanwhile, forced expression of WTAP caused an inverse phenotype in xenograft mice (Fig. 2h-j; Additional file 8: Figure S2b-d). In summary, we believed that WTAP performed a tumor promoting function in HCC.

### ETS1 was identified as the potential target of WTAP in HCC

**Fig. 3:**
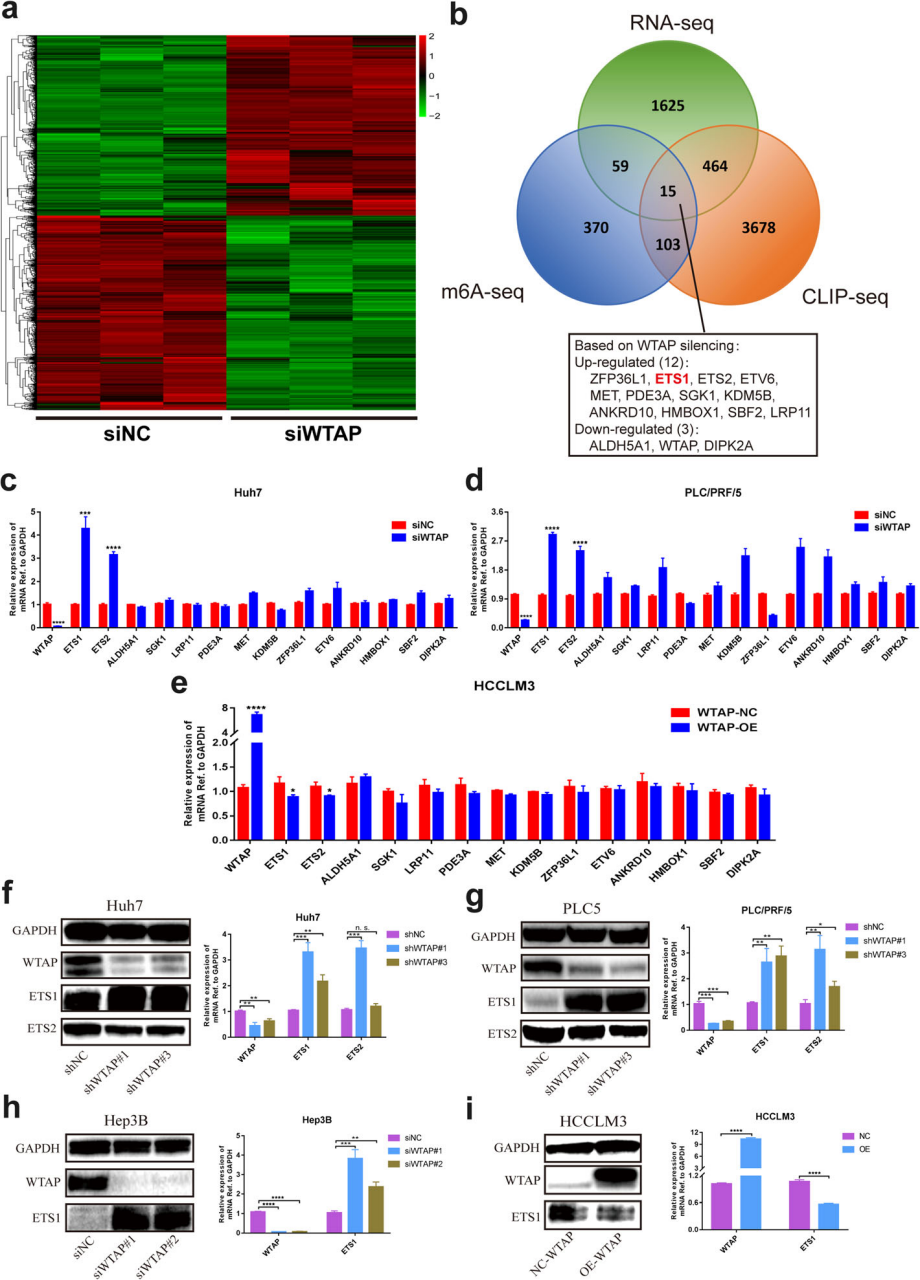
A high-throughput sequencing combination revealed ETS1 to be the target of WTAP. **a** Transcriptome profiles from Huh7 cells transfected with the WTAP siRNAs or negative control siRNAs (both in triplicate) were shown. Bands with red, black or green in the heat map indicated high, moderate or low expression, respectively. **b** A Venn diagram was generated from the gene sets enriched for transcripts that were substantially altered after WTAP silencing (RNA-seq), along with those enriched for m6A-modified transcripts (m6A-seq) and those enriched for WTAP-conjugated transcripts (CLIP-seq). 15 genes were selected according to the overlaps. The RNA-seq data was acquired from our study, while the m6A-seq and CLIP-seq data were obtained from GEO datasets (GSE46705). Information regarding detailed gene sets of RNA-seq was listed in Additional file 4: Table S4; **c**, **d** and **e** RT-qPCR was performed in Huh7 (**c**) and PLC/PRF/5 (**d**) with WTAP silencing, and in HCCLM3 (**e**) with WTAP overexpression, to validate the overlapped genes. The variation of ETS1 and ETS2 was consistent among 15 genes in the above three cell lines; **f** and **g** Expression of ETS1 and ETS2 following WTAP knockdown was evaluated by western blotting and RT-qPCR in Huh7 (**f**) and PLC/PRF/5 cells (**g**); **h** and **i** Expression of ETS1 was further examined by western blotting and RT-qPCR in Hep3B (**h**) or HCCLM3 (**i**) cells following the knockdown or overexpression of WTAP

To understand the underlying mechanism by which WTAP exerted tumor promoting effects in HCC, we employed transcriptome sequencing to illustrate the transcriptional alterations in WTAP knockdown cells. Hierarchical clustering indicated 1636 up-regulated genes and 794 down-regulated genes after the deletion of WTAP (Fig. 3a). In order to narrow down the scope of downstream targets, we integrated the GEO data (GSE46705) published by Liu et al. [40] who performed MeRIP -seq and CLIP-seq for elaborating the transcripts regulated by WTAP, to establish the gene set overlaps. A total of 15 genes were unveiled by the Venn diagram (Fig. 3b). Then we conducted RT-qPCR to evaluate the impact of WTAP on each candidate. Among which, ETS1 and ETS2 were consistently significantly up-regulated following WTAP silencing, and they were both moderately down-regulated when WTAP was overexpressed (Fig. 3c-e). However, western blotting analyses revealed that the inactivation of WTAP notably led to the elevation of ETS1 at the protein level, rather than ETS2, (Fig. 3f, g). We further demonstrated that the ETS1 content was conversely affected by WTAP in Hep3B and HCCLM3 cells (Fig. 3h, i), accompanied by the parallel results from immunofluorescence staining and cytosolic/nuclear separation analysis (Additional file 9: Figure S3a, b). Therefore, we postulated that dysfunction of ETS1 probably accounted for the WTAP-mediated HCC proliferation signature.

### WTAP increased m6A modification of ETS1 mRNA

**Fig. 4:**
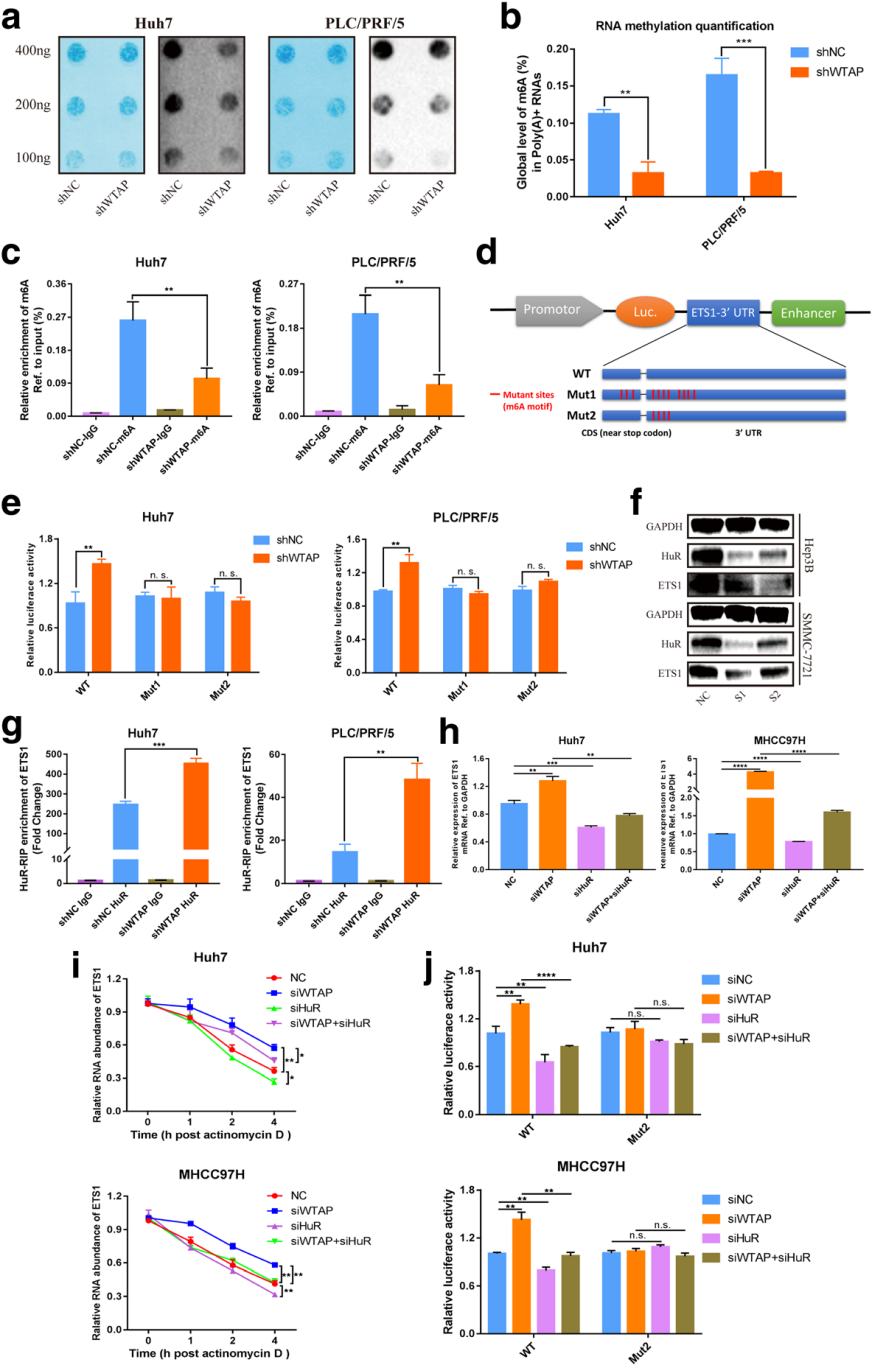
WTAP repressed ETS1 in an m6A-HuR mediated pattern. **a** The m6A level of poly(A) + RNAs isolated from total RNA of WTAP-knockdown Huh7 and PLC/PRF/5 cells was indicated by m6A dot blot. Corresponding RNAs were loaded equally by a 2-fold serial dilution with 400 ng, 200 ng and 100 ng. Methylene blue staining served as a loading control; **b** The global content of m6A was also examined by RNA methylation quantification assay, relying on the standard curve; **c** MeRIP analysis followed by qRT-PCR was applied to assess the m6A modification of ETS1 in two WTAP-silencing HCC cells. The enrichment of m6A in each group was calculated by m6A-IP/input and IgG-IP/input. **d** Three luciferase plasmids were constructed by inserting the corresponding cDNAs into pGL3-control vectors. Wild-type reporters embodied the full-length 3’UTR and a partial CDS sequence near stop codon of ETS1 with intact m6A sites, while mutant ones obtained some A-C mutations on m6A consensus motifs (Mut1 or Mut2 contained 11 or 4 mutations, respectively). Luciferase activity was detected and normalized to Renilla activity; **e** Relative activity of the WT or Mut luciferase reporters in WTAP-silenced Huh7 and PLC/PRF/5 cells was determined (normalized to negative control groups); **f** ETS1 expression was identified by western blotting in Hep3B and SMMC-7721 cells upon knockdown of HuR (#1, #2) compared with siNC; **g** Immunoprecipitation of HuR-related RNA in control or WTAP-knockdown cells was conducted followed by RT-qPCR to detect the amount of ETS1 mRNA binding to HuR; **h** ETS1 expression was measured by RT-qPCR in Huh7 and MHCC97H cells with or without knockdown of WTAP or HuR compared with NC; **i** The RNA decay rate was determined in Huh7 and MHCC97H cells after treatment with Actinomycin D (normalized to 0 h); **j** The relative activity of the WT or Mut luciferase reporters was detected in WTAP/HuR-rescued Huh7 and MHCC97H cells (normalized to negative control groups)

According to the MeRIP-seq data, modulation of ETS1 might occur in an m6A-dependent manner. To determine whether the m6A modification of ETS1 was mediated by WTAP, we first measured the global level of m6A in negative control group and stable WTAP knockdown group through two distinct methods (m6A dot blot and RNA methylation quantification assay). As expected, m6A levels were substantially decreased with the deletion of WTAP in two HCC cell lines (Fig. 4a, b). Furthermore, the MeRIP-qPCR assay was conducted to determine the enrichment of m6A in ETS1. Compared with IgG control, m6A-specific antibody robustly enriched ETS1 transcripts. Furthermore, we found a remarkably decreased amount of ETS1 modified by m6A following WTAP silencing (Fig. 4c). Therefore, we supposed that WTAP was able to influence the overall level of m6A, specifically for ETS1.

To substantiate the requirement of m6A modification for ETS1, luciferase reporter assays were conducted with a wild-type (WT) and two mutant (Mut) plasmids. For Mut reporters, several adenosine bases (A) in predicted m6A sites were replaced by cytosine bases (C) to eliminate the effect of m6A methylation, while WT reporter contained intact m6A sites (Fig. 4d). As expected, the luciferase activity of WT group moderately intensified under WTAP knockdown, while those of Mut groups definitely rendered resistance to the impact of WTAP silencing (Fig. 4e), implying that the regulation of ETS1 was under the control of WTAP-guided m6A modification.

Intriguingly, the direct protein interaction of WTAP and ETS1 seemed to be unwarranted (Additional file 9: Figure S3c). In addition, the IF staining failed to identify the colocalization of WTAP and ETS1, because WTAP was primarily located in the nucleus while ETS1 was mainly in the cytoplasm (Additional file 9: Figure S3a, b), which was in accordance with previous studies [9, 41]. Nevertheless, the WTAP antibody could lead to the enrichment of ETS1 mRNA (Additional file 9: Figure S3d), which was in agreement with the CLIP-seq data. Therefore, we further confirmed that the association of WTAP and ETS1 may not be at the protein level, but rather the RNA level.

To further elucidate the effect of m6A in regulation of ETS1, we introduced the methylation inhibitors, 3-deazaadenosine (DAA) and cycloleucine. Consistent with our hypothesis, treating HCC cells with DAA or cycloleucine led to a remarkable reduction of total m6A level (Additional file 9: Figure S3e), and markedly increased the expression of ETS1 meanwhile (Additional file 9: Figure S3f, g).

Collectively, these data implied that WTAP was sufficient to regulate the m6A modification of ETS1 mRNA.

### WTAP suppressed expression of ETS1 in an m6A-HuR mediated manner

It was indispensable to find appropriate readers of ETS1, since m6A-modified transcripts relied on the effector proteins to be functionally involved in biological processes. As the well-known m6A reader protein, YTHDF2 was reported to frequently participate in the regulation of mRNA degradation [18]. However, knockdown of YTHDF2 caused little impact on ETS1 expression (Additional file 10: Figure S4a), which precluded the potential role of YTHDF2 in ETS1 modulation. In addition, the RNA-binding protein (RBP) HuR was prone to binding with less m6A-modified RNA and stabilizing its bound counterparts [42], hence we supposed that HuR may regulate ETS1. Actually, the reduction of HuR prominently alleviated the expression of ETS1 (Fig. 4f). And we found that HuR-specific antibody dramatically enriched ETS1 mRNA compared to the IgG control, while repression of WTAP significantly intensified the enrichment of ETS1 mRNA as manifested by RIP-qPCR (Fig. 4g). Interestingly, HuR did not physically interact with WTAP according to Co-IP results (Additional file 9: Figure S3c), and HuR was rarely altered when WTAP was silenced (Additional file 10: Figure S4b, c). This signified that WTAP restrained ETS1 via interference of the conjunction between HuR protein and ETS1 mRNA, instead of altering the expression of HuR.

Moreover, the elevation of ETS1 induced by WTAP knockdown could be retrieved by attenuation of HuR (Fig. 4h and Additional file 10: Figure S4d). We found knockdown of WTAP would prolong the half-life of ETS1 RNA, while HuR silencing could reverse this effect (Fig. 4i). Additionally, we carried out luciferase reporter assays to determine the involvement of HuR in m6A modification. In WT groups, the enhancement of luciferase activity induced by WTAP silencing could be rescued by HuR. However, alteration of HuR expression seemed to be ineffective for luciferase activity when possible m6A sites of ETS1 were mutated (Fig. 4j).

Together, our findings suggested that WTAP repressed ETS1 through an m6A-HuR-dependent pathway.

### ETS1 served as a tumor suppressor and reversed the effects of WTAP in HCC

**Fig. 5:**
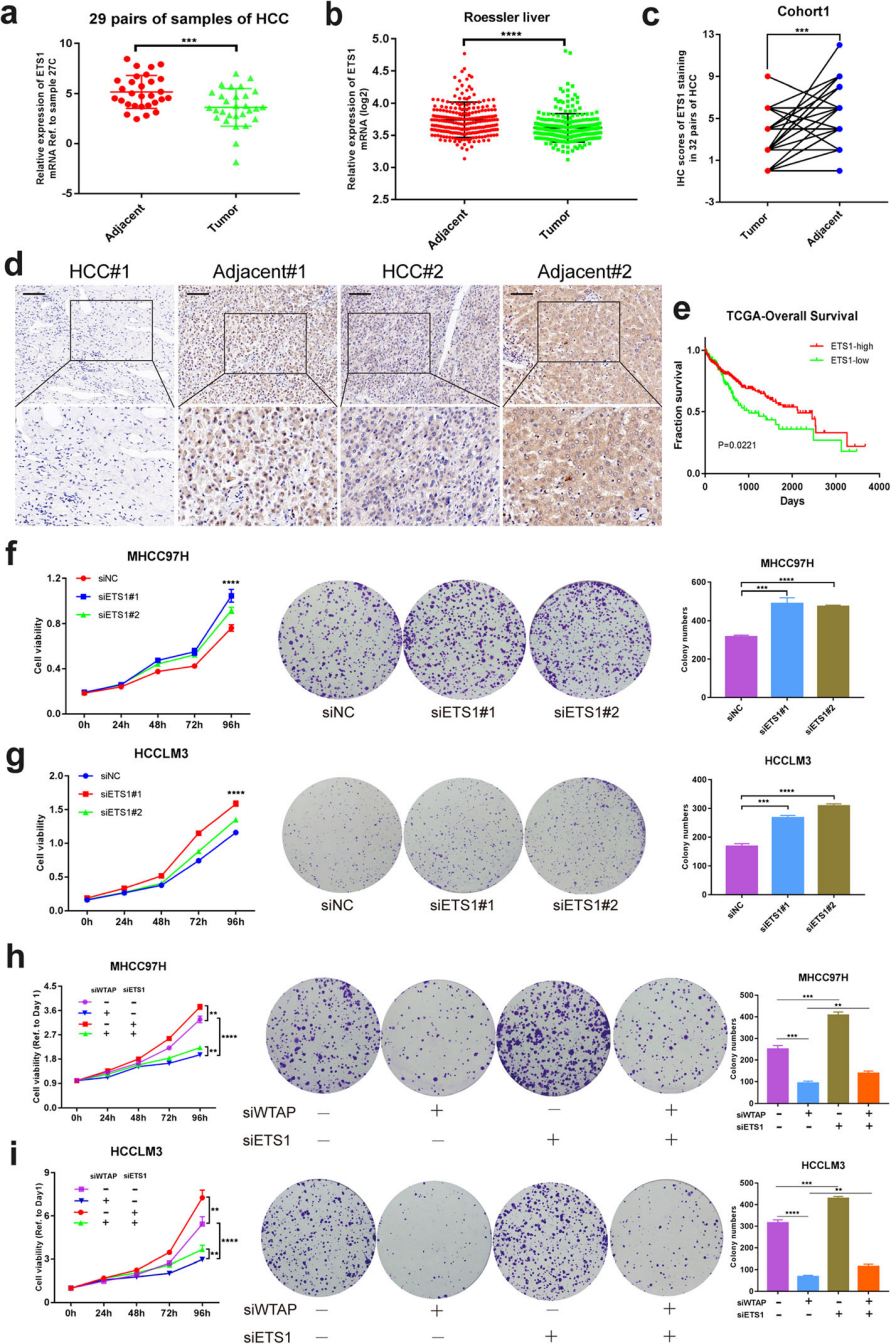
ETS1 played a tumor suppression role in HCC via the reversal of phenotypes mediated by WTAP. **a** Expression of ETS1 in HCC tumor and adjacent tissues from 29 pairs of HCC samples; **b** Expression of ETS1 mRNA was demonstrated based upon GEO datasets (GSE14520); **c** IHC scores of 32 pairs of HCC tissues in cohort1 based on ETS1 staining; **d** Representative IHC images of ETS1 staining in HCC tumor or adjacent tissues (scale bar, 100 μm; magnification, 200X and 400X); **e** Overall survival of HCC patients according to the level of ETS1 (data from TCGA); **f** and **g** CCK8 and colony formation assays were performed to examine the propagation ability of MHCC97H (**f**) and HCCLM3 (**g**), where ETS1 was knocked down or not, with bar charts indicating the colony numbers (right panel); **h** and **i** Rescue experiments were conducted to determine the influence of ETS1 silencing on WTAP knockdown cells (MHCC97H and HCCLM3), with bar charts showing colony numbers (right panel)

Expression of ETS1 in tumor tissues was identified to be lower compared with paratumor tissues in HCC (Fig. 5a, b). Moreover, we discovered a markedly higher positive rate in adjacent tissues based on the IHC staining of cohort1 (Fig. 5c, d). And high level of ETS1 implied better prognosis according to the TCGA data (Fig. 5e). Functional validation in vitro including CCK-8 and colony formation demonstrated that knockdown of ETS1 enhanced the proliferation capability of MHCC97H, HCCLM3 and Huh7 cell (Fig. 5f, g and Additional file 11: Figure S5a). Moreover, ETS1 silencing was sufficient to rescue the inhibitory effect of WTAP knockdown on HCC cell growth and viability (Fig. 5h, i). Hence the inactivation of ETS1 may lead to the progression of HCC via the WTAP-ETS1 axis.

### Silencing of WTAP caused G2/M arrest in HCC through the ETS1-p21/p27 axis

**Fig. 6:**
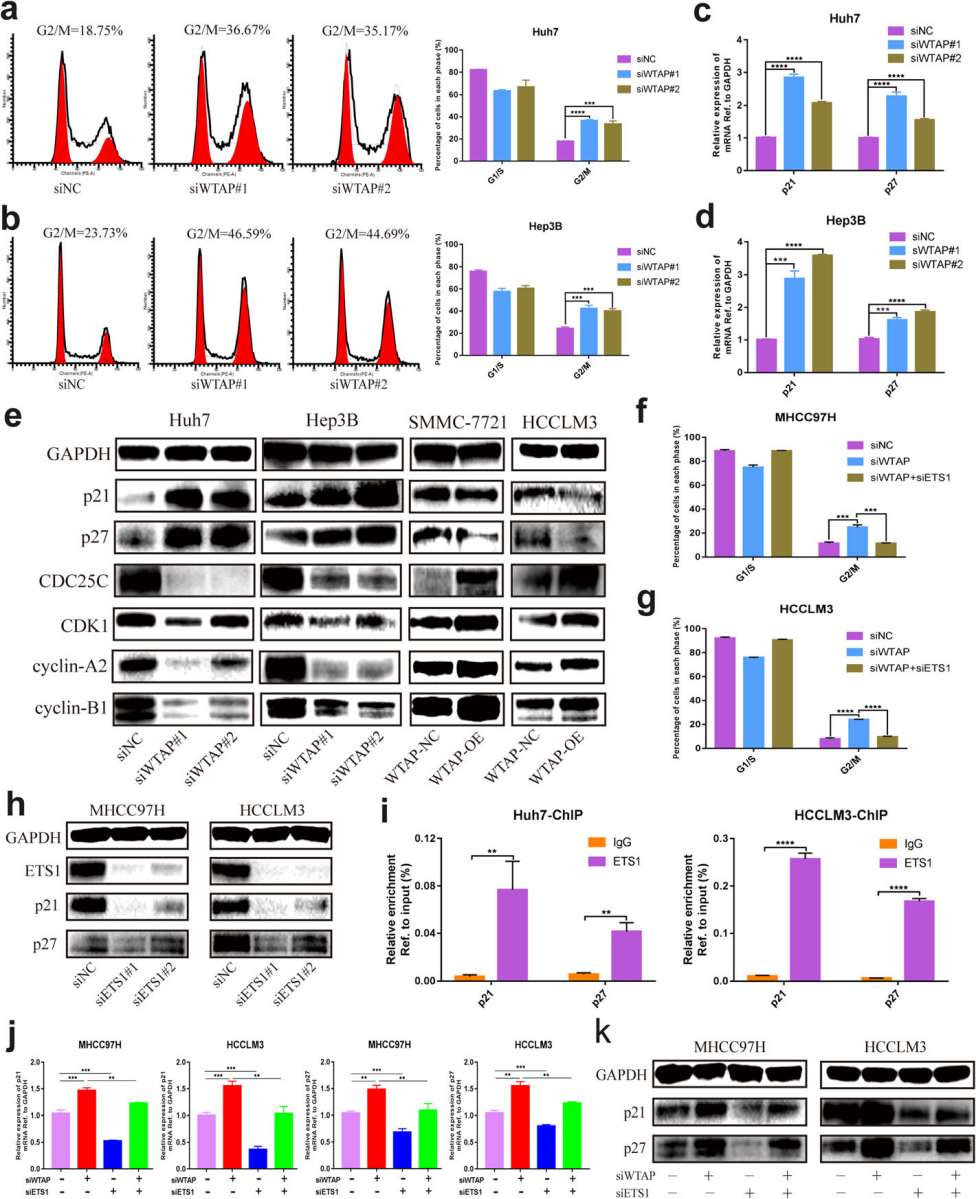
WTAP was involved in the cell cycle by alleviating the expression of p21 and p27. **a** and **b** Cell cycle distribution was analyzed by flow cytometry in Huh7 (**a**) and Hep3B (**b**) cells where WTAP were silenced, with summary bar charts showing the percentage of cells in each phase; **c** and **d** RT-qPCR was utilized to explore alterations of p21 and p27 when WTAP was knocked down in Huh7 (**c**) and Hep3B (**d**), respectively; **e** Cell cycle-related proteins including p21, p27, CDC25C, CDK1, cyclin-A2 and cyclin-B1 were measured by western blot in the indicated cells where WTAP was knocked down (Huh7 and Hep3B) or overexpressed (SMMC-7721 and HCCLM3); **f** and **g** Flow cytometry indicated that ETS1 knockdown could reverse the G2/M arrest in WTAP-silenced MHCC97H (**f**) or HCCLM3 (**g**) cell; **h** The knockdown efficiency of ETS1 was verified followed by the detection of p21 and p27 expression via western blotting; **i** The ChIP assay was conducted in Huh7 and HCCLM3 cells to determine whether ETS1 could bind to the promoter of p21 and p27 (IP/input was calculated); **j** and **k** A rescue assay was performed with or without knockdown of WTAP or ETS1 to validate the retrieved role of ETS1 in WTAP-mediated events. Expression of p21 and p27 were detected at the RNA (j) and protein (**k**) levels

WTAP was reported to be involved cell cycle regulation, thus we intended to explore the related mechanism in HCC. In accordance with the results above, WTAP knockdown caused substantial G2/M arrest (Fig. 6a, b), while overexpression moderately contributed to the promotion of G2/M phase transition (Additional file 11: Figure S5b). To clarify the underlying mechanism, we examined several elements associated with the cell cycle. Intriguingly, the expression of p21 and p27, the tumor suppressors that played vital roles in cell cycle and proliferation, were strongly increased when WTAP was inactivated at transcriptional level (Fig. 6c, d and Additional file 11: Figure S5c). Meanwhile, western blotting analysis identified that WTAP knockdown upregulated p21 and p27, and simultaneously downregulated CDC25C, CDK1, cyclin-A2 and cyclin-B1 (Fig. 6e). In contrast, overexpression of WTAP alleviated the expression of p21 and p27 and reduced the magnification of all the indicated checkpoint proteins in the G2 phase (Fig. 6e).

To further investigate whether ETS1 participated in WTAP-induced effects on cell cycle, we conducted a series of rescue assays. Although ETS1 deficiency did not independently intervene in the cell cycle distribution (Additional file 11: Figure S5d, e), the G2/M arrest vcaused by WTAP silencing was significantly inversed by suppression of ETS1 (Fig. 6f, g; Additional file 12: Figure S6a, b). And WTAP deficiency followed by ETS1 overexpression led to a more remarkable G2/M arrest (Additional file 12: Figure S6c, d). Additionally, knockdown of ETS1 induced the decrease of p21 and p27 (Additional file 12: Figure S6e), and western blotting analysis revealed the same conclusion (Fig. 6h). We speculated that ETS1 may enhance the transcription of p21 and p27 since the ChIP assay indicated that ETS1 could bind to their promoter (Fig. 6i). Moreover, the repression of ETS1 reinstated the elevated expression of p21 and p27 caused by WTAP inhibition (Fig. 6j, k), while epitopic expression of ETS1 contributed to further up-regulation of p21 and p27 upon WTAP silencing (Additional file 12: Figure S6f). These data suggested ETS1-p21/p27 axis was essential for WTAP-dependent cell cycle regulation in HCC.

### Combination of high WTAP and low ETS1 expression predicted unfavorable outcomes of HCC

**Fig. 7:**
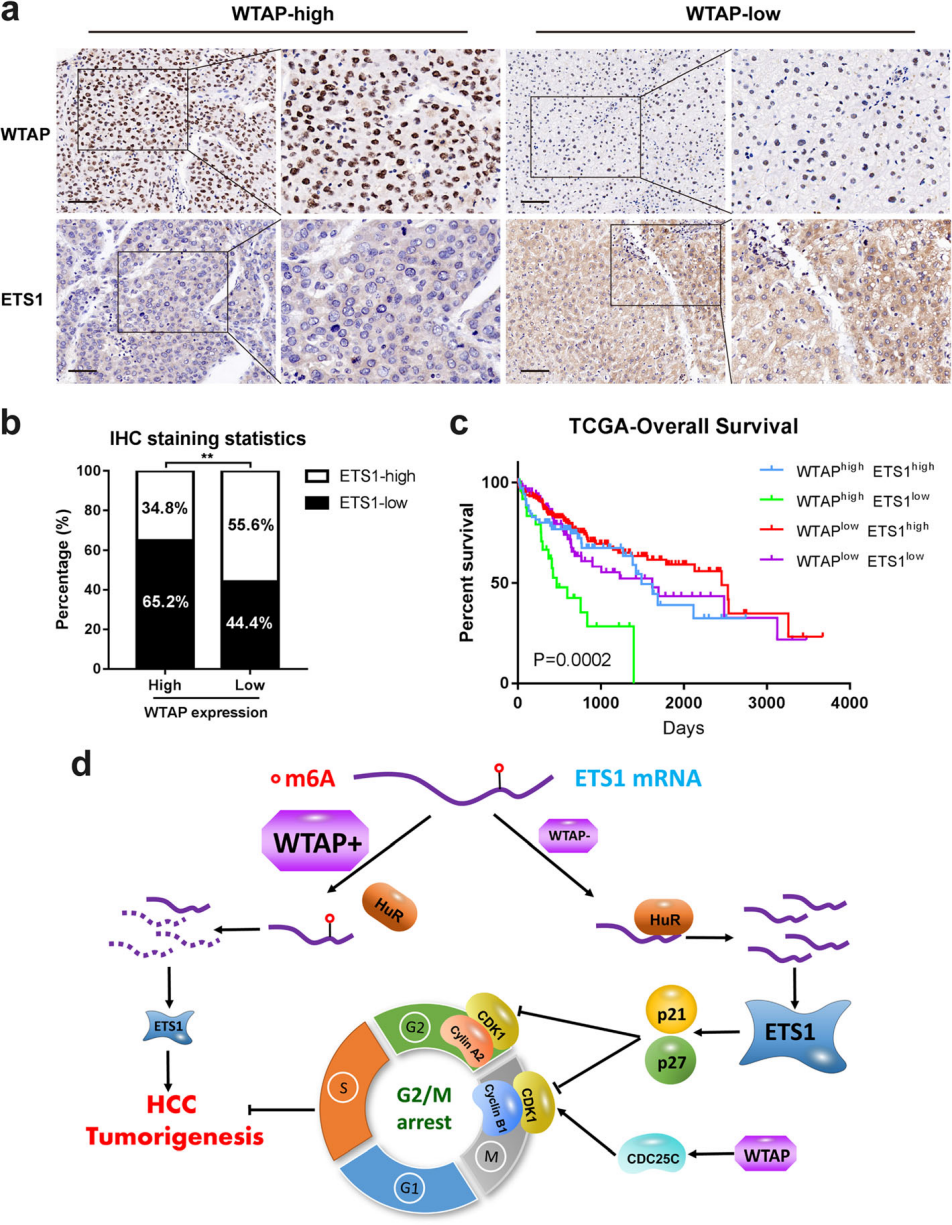
High WTAP expression was correlated with low ETS1 expression and revealed a poor prognosis of HCC. **a** Representative IHC staining images of the identical HCC specimens with the staining of WTAP or ETS1 were shown, respectively (cohort1) (scale bar, 100 μm; magnification, 200X and 400X); **b** IHC results revealed that expression of WTAP and ETS1 was negatively interrelated in HCC tissues; **c** Overall survival analysis based on the co-expression of WTAP and ETS1 in HCC according to TCGA data; **d** A schematic model illustrating our findings on WTAP-mediated m6A regulation was shown

To evaluate the clinical correlation between WTAP and ETS1, we performed IHC assays of WTAP and ETS1 staining within the same HCC specimens from cohort1. Nearly 65.2% of samples where WTAP was more highly expressed presented weaker ETS1 staining, while approximately 55.6% of those with lower WTAP expression exhibited stronger ETS1 staining (Fig. 7a, b). We also confirmed that high WTAP expression or low ETS1 expression was independently associated with poor prognosis of HCC patients (Figs. 1e, f and 5e). Then the Kaplan-Meier analysis based on the combination of these two elements further demonstrated that HCC individuals with the expression of WTAP^high^ETS1^low^ had an even worse overall survival rate than any other groups (*P* = 0.0002), especially compared with those who were in the state of WTAP^low^ETS1^high^ (Fig. 7c). To conclude, WTAP and ETS1 were inversely interrelated in clinical samples and the co-expression pattern of WTAP and ETS1 might be regarded as an efficient prognostic factor of HCC.

## Discussion

As a recent hot topic in the area of epidemic regulation, RNA m6A modification is involved in multiple cellular processes such as mRNA maturation, protein translation and molecular structure switching [43]. Accumulating evidence indicates that the dysregulation of m6A profoundly contributes to the pathogenesis of various diseases, including HCC [44]. We examined the expression of four m6A methyltransferases in HCC samples, and found METTL3, WTAP and KIAA1429 were all overexpressed in tumors compared with adjacent tissues (Fig. 1b and Additional file 13: Figure S7a, b). Indeed, METTL3 and METTL14 have been clarified to affect the proliferation ability and metastatic potential of HCC cells via distinct mechanism [29, 30], whereas the role of WTAP in HCC still remains obscure. In our present study, we first identified that the elevated WTAP expression was accompanied by a poor HCC prognosis. Based on the transient and stable WTAP knockdown or forced expression cells, we then functionally delineated that WTAP facilitated HCC growth and invasion in vitro and in vivo. Mechanistically, we confirmed that ETS1, which was subsequently defined to be a suppressor in HCC, was modulated by WTAP in an m6A-mediated and HuR-containing manner. Furthermore, WTAP was proved to regulate the cell cycle of HCC cells in a p21/p27-dependent pattern with the involvement of ETS1.

Actually, WTAP was overexpressed and served as a considerable risk factor in a variety of tumors [35–39]. Similar conclusions were obtained according to the analysis of TCGA pan-cancer data (Additional file 13: Figure S7c-j). Some WTAP-related pathways have been proposed such as EGF signaling [36], the mTOR pathway or the WT1-TBL1 axis [45], while the involvement of WTAP as an m6A regulator in human cancers has not been explored previously. In our study, both m6A dot blot and RNA methylation quantification assays implied that WTAP was essential for m6A modification since the overall m6A level dramatically declined upon WTAP deficiency (Fig. 4a, b). To further address the role of WTAP, we combined the data from RNA-seq, m6A-seq and CLIP-seq to reveal that ETS1 was the downstream target of WTAP. ETS1 was inversely regulated by WTAP and was modified in the 3′ UTR by WTAP-intermediated m6A methylation as determined by MeRIP-qPCR and luciferase reporter assay (Fig. 4c-e), followed by identification with methylation inhibitors (Additional file 9: Figure S3e-g). Thus, WTAP may participate in tumor progression functioning as an m6A mediator.

Notably, reader proteins were pivotal for m6A modification to exert diverse biological functions, while YTHDF2-dependent m6A post-transcriptional suppression was a typical module [18]. However, the decay of ETS1 mRNA seemed not to be affected by YTHDF2 (Additional file 10: Figure S4a). Additionally, in contrast to YTHDF2, the well-established RNA stabilizer HuR was reported to immobilize demethylated mRNA by blocking miRNA binding in 3′ UTRs [42]. We performed HuR-RIP and observed an increase in HuR binding to ETS1 mRNA when WTAP was silenced (Fig. 4g). Importantly, this was not caused by the direct impact of WTAP on HuR expression (Additional file 10: Figure S4b, c), but was probably induced by the demethylation with the deletion of WTAP. Generally, HuR was recognized as an indirect m6A effector with a preference for less m6A-modified transcripts [26, 42, 46]. Nevertheless, there are also discrepant findings challenging this assumption. Huang et al. showed that IGF2BPs recruited HuR to protect m6A-containing RNAs [24], while Visvanathan et al. argued that m6A modification and HuR complementarily attributed to RNA stabilization based on an analysis of cumulative expression distribution [47]. These controversial results may underscore the complicated character of HuR in m6A-modulation which requires further illustrations.

As the first member in ETS family, ETS1 has primarily been described as a transcriptional activator typically regulated by Ras/Raf/MEK/ERK pathway [48]. It was prevalently overexpressed in carcinomas including breast, colorectal, gastric, lung cancer, etc. [49] However, few studies had attempted to identify the biological role of ETS1 in HCC. Ito et al. [50] reported that although higher expression of ETS1 was detected in tumor tissues compared with normal liver, ETS1 was remarkably up-regulated in noncancerous lesions compared with HCC lesions, which emphasized the special character of ETS1 in liver tumorigenesis. Consistently, lower expression of ETS1 was found in HCC tissues with worse outcomes for overall survival (Fig. 5a-e). In addition, we functionally determined that silencing of ETS1 contributed to impaired viability and proliferation capability of two HCC cells (Fig. 5f, g). Similarly, a repressive effect of ETS1 based on soft agar assays was illustrated in breast cancer cell growth [51], collectively denoting that the influence of ETS1 on cellular growth depended on tissue context and tumor type. Actually, post-transcriptional regulation was vital for ETS1 including phosphorylation, ubiquitination, sumoylation and acetylation [49]. We have now determined that ETS1 could be altered by m6A RNA methylation as well, at least mediated by WTAP.

Besides, also belonging to ETS family, ETS2 was revealed via sequencing to be another potential target of WTAP (Fig. 3a, b), a situation that was intensely analogous to ETS1. As indicated by MeRIP-qPCR assay, ETS2 was modified by the WTAP-related m6A methylation in the 3′ UTR (Additional file 14: Figure S8c, d). And the evidence from methylation inhibitors supported this hypothesis as well (Additional file 14: Figure S8e-g). Additionally, YTHDF2-RIP and knockdown of YTHDF2 revealed that the decay of ETS2 may occur in an YTHDF2-dependent manner (Additional file 14: Figure S8h, i). Similar to ETS1, ETS2 was recognized as a depressor in HCC according to its basal expression, survival analysis and functional assays (Additional file 15: Figure S9a-e). Surprisingly, we were regretful to find that ETS2 was conversely regulated by WTAP merely at the RNA level, but not the protein level (Fig. 3c-g and Additional file 14: Figure S8a, b). Therefore, the WTAP-m6A-YTHDF2-ETS2 pathway was in doubt. Herein, we postulated that perhaps not all of the m6A modification was meaningful even though the sites were accurately defined, since other more complicated regulation mechanisms may be involved in. Future work is demanded to delineate which subsets of m6A methylation is functionally involved in corresponding biological processes.

Furthermore, WTAP was identified to be moderately involved in cell cycle regulation. We currently verified that WTAP deficiency induced a conspicuous accumulation of G2 phase (Fig. 6a, b), which was in line with the data of Horiuchi et al. [32]. It was reported that the stability of cyclin A2 and CDK2 mRNA regulated by WTAP contributed to the variation of mitotic cycle transition [32, 35]. We then investigated which proteins were responsible for the G2/M arrest by examining G2 phase-associated signaling. P21 and p27 were negatively modulated by WTAP, whereas the downstream signaling including CDC25C, CDK1, cyclin A2 and cyclin B1 presented the opposite alternations (Fig. 6c-e). Indeed, ETS1 could motivate the transcription of p21 by binding to its promoter [52], which was also supported by our findings (Fig. 6h, i and Additional file 12: Figure S6e). Furthermore, p27 was validated to be likewise controlled by ETS1 (Fig. 6h, i and Additional file 12: Figure S6e). Moreover, ETS1 inhibition abolished the elevated expression of p21 or p27 induced by WTAP silencing (Fig. 6j, k), while overexpression of ETS1 further promoted their up-regulation (Additional file 12: Figure S6f). Overall, we provided novel insights into the WTAP-mediated cell cycle modulation via ETS1-p21/p27 axis in HCC.

Although we reported the WTAP-dependent tumorigenesis of HCC here for the first time, there are still several limitations to our study. To reveal the downstream target of WTAP, we employed m6A-seq and CLIP-seq results from the GEO data, which were partly based on Hela cells, not HCC cells. Therefore, we recognize that these transcripts enriched for m6A modification may not be sufficient and we might have omitted some m6A-associated candidates specifically involved in HCC which deserves further investigations. Additionally, whether the m6A modification of ETS1 prevails in various cell types requires our validation. Besides, the in vivo data should be extended to strengthen our hypothesis, such as the introduction of patient-derived tumor xenograft (PDX) models. Finally, the possibility that WTAP may function in a detrimental role beyond m6A-related mechanisms in HCC progression ought to be explored.

## Conclusions

In summary, our study has illustrated the oncogenic role of WTAP and an activated WTAP-mediated m6A machinery in human HCC. WTAP up-regulation contributes to the m6A modification of ETS1 followed by epigenetic silencing of ETS1 via a HuR-associated manner. Our findings enrich the functional value of m6A methylation in hallmarks of tumors, and open up potential avenues for exploring efficient therapeutic strategies in the treatment of HCC.

## Additional files

Additional file 1: **Table S1.** Sequences of primers used in this study. (XLSX 9 kb)

Additional file 2: **Table S2.** Antibodies used in this work. (XLSX 9 kb)

Additional file 3: **Table S3.** Target sequences of siRNAs or shRNAs used in this work. (XLSX 8 kb)

Additional file 4: **Table S4.** RNA-seq results in this work. (XLSX 3149 kb)

Additional file 5: **Data S1.** Potential m6A sites of ETS1 and related primers in this work. (DOCX 16 kb)

Additional file 6: **Data S2.** Sequences of wild-type or mutant reporter plasmids in this work. (DOCX 15 kb)

Additional file 7: **Figure S1.** Expression of WTAP in cell lines and functional investigations of WTAP. a Expression and survival analysis of WTAP in HCC (data from TCGA, analyzed with UALACN, http://ualcan.path.uab.edu/analysis.html); b, c mRNA (b) and protein (c) level of WTAP in an immortalized hepatic cell line (QSG-7701) and nine HCC cell lines; d, e Negative control vector or Flag-WTAP was transfected into HCCLM3 (d) or SMMC-7721 (e) with the overexpression efficiency determined. Proliferation capacities were detected by CCK-8, colony formation assay; f, g Representative images and bar charts of cell migration and invasion ability in Huh7 cells with WTAP knockdown (siRNA or shRNA) or negative control detected by transwell and matrigel transwell assays (scale bar, 100 μm). (TIF 3566 kb)

Additional file 8: **Figure S2.** The impact of WTAP in vivo*.* a The level of WTAP and Ki67 in xenograft tumor tissues was detected by IHC (scale bar, 50 μm; magnification, 400X); b-d Tumor growth curve (c) of SMMC7721 with stable WTAP epitopic expression cells in a xenograft mouse model was based on the tumor sizes. And the photography (b) and tumor weights (d) were recorded to exhibit the growth difference within the influence of WTAP. (TIF 4248 kb)

Additional file 9: **Figure S3.** Mechanisms of WTAP-mediated modulation on ETS1. a Representative immunofluorescence images of Huh7 cells with the deficiency of WTAP to determine the subcellular distribution and expression of WTAP and ETS1 (scale bar, 30 μm). WTAP mainly localized in nucleus while ETS1 mainly in cytoplasm. However, fluorescence intensity of ETS1 significantly augmented in either cytosolic or nuclear regions (especially in nuclear membrane); b Cytosolic and nuclear separation analysis was conducted to examine the expression of ETS1 within subcellular components under WTAP silencing; c The protein interaction between WTAP and ETS1 or HuR was precluded by Co-IP assay; d WTAP-RIP was applied to verify the enrichment of ETS1 mRNA by WTAP antibody; e Overall level of m6A was determined by RNA methylation quantification assay after the treatment of DAA and cyclolencine in Huh7 cell with diverse concentration, respectively; f and g Huh7 and PLC/PRF/5 was treated with DAA in the concentration of 0uM, 100uM, 200uM; Another panel, was treated with cyclolencine in the concentration of 0 mM, 50 mM, 100 mM. And the expression of ETS1 was detected in RNA (f) and protein (g) level. (TIF 1651 kb)

Additional file 10: **Figure S4.** Mechanisms of HuR-involved regulation of ETS1. a YTHDF2 was knockdown in PLC/PRF/5 without any variation in ETS1 expression; b and c WTAP was knockdown followed by qRT-PCR (b) and western blotting (c) to estimate the alteration of HuR; d WTAP-inactivation caused a striking enlargement of ETS1, which could be rescued by knockdown of HuR. (TIF 755 kb)

Additional file 11: **Figure S5.** Proliferation and cell cycle investigations of ETS1. a CCK8 and colony formation assay were performed to test propagation ability of Huh7 cell where ETS1 was knockdown; b Cell cycle distribution was analyzed by flow cytometry in SMMC-7721 cell where WTAP was overexpression, with bar charts indicating the percentage of cells in each phase; c RT-qPCR was used to find changes of p21 and p27 when WTAP was knockdown in PLC/PRF/5; d and e Flow cytometric analysis was conducted in MHCC97H (d) and HCCLM3 (e) cell with the inactivation of ETS1. (TIF 1434 kb)

Additional file 12: **Figure S6.** Cell cycle inquiry in WTAP/ETS1 rescued cells a and b Rescue assays of cell cycle distribution were performed in WTAP-silenced MHCC97H (a) and HCCLM3 (b) cells with or without siETS1; c and d Cell cycle distribution were performed in WTAP-silenced Huh7 (c) and MHCC97H (d) cells with or without ETS1 overexpression; e Expression of p21 or p27 was measured with the reduction of ETS1 in RNA level; f Expression of p21 and p27 was measured in WTAP-knockdown Huh7 and MHCC97H cells with or without ETS1 overexpression. (TIF 1000 kb)

Additional file 13: **Figure S7.** Pan-cancer expression and survival analysis of WTAP and other m6A-related enzymes. a Expression of METTL3, METT14 and KIAA1429 protein was analyzed by western blotting in 15 pairs of HCC tissues; (T: tumor; P: peritumor); b Expression and survival analysis of three enzymes mentioned above (data from TCGA, analyzed with UALACN); c-f Expression of WTAP in tumor and para-tumor tissues of Colon adenocarcinoma (c), Lung squamous cell carcinoma (d), Stomach adenocarcinoma (e) and Head and Neck squamous cell carcinoma (f) (data from TCGA, analyzed with UALACN); g-j Overall survival curves of HCC patients according to the expression of WTAP in four tumors (data from TCGA, analyzed with KM plotter, http://kmplot.com/analysis/). (TIF 1805 kb)

Additional file 14: **Figure S8.** ETS2 was negatively regulated by WTAP via m6A-YTHDF2 pathway. a Expression of ETS2 was measured following silencing of WTAP in HCCLM3 cell; b A retrieval assay was performed with the knockdown of WTAP or ETS2; c, d WTAP-mediated m6A modification of ETS2 was assessed by MeRIP-qPCR with specific primers; e, f Expression of ETS2 after treatment of DAA (e) and cyclolencine (f) by RT-qPCR; g Expression of ETS2 after treatment of DAA was detected by western blotting; h YTHDF2-RIP was applied to evaluate the enrichment of ETS2 by YTHDF2 antibody; i Expression of ETS2 was surveyed upon the knockdown of YTHDF2. (TIF 827 kb)

Additional file 15: **Figure S9.** Expression, survival and functional analyses of ETS2. a Expression of ETS2 in HCC cancerous and normal tissues from 29 pairs of HCC samples; b Expression of ETS2 in HCC cancerous and normal tissues based on TCGA datasets; c Overall survival of HCC patients grouped by the level of ETS2; d CCK-8 and colony formation assays was applied to determine the viability of ETS2 knockdown cell; e Transwell assays was utilized to evaluate the motility of ETS2 knockdown cells (scale bar, 100 μm). (TIF 3161 kb)

## Data Availability

All data generated or analyzed during the current study are included in this published article (and its supplementary information files) or available on published datasets (TCGA or GEO).

## Abbreviations

5AZA: 5-Aza-2′-deoxycytidine
AFP: Alpha-fetoprotein
AML: Acute myeloid leukaemia
CCK-8: Cell Counting Kit-8
ChIP: Chromatin immunoprecipitation
CI: Confidence interval
Co-IP: Co-Immunoprecipitation
DAA: 3-Deazaadenosine
DAPI: 4′,6-diamidino-2-phenylindole
DFS: Disease-free survival
EdU: 5-ethynyl-20-deoxyuridine
ETS1: ETS proto-oncogene 1
GEO: Gene expression omnibus
HCC: Hepatocellular carcinoma
HR: Hazard ratio
HuR: Hu-Antigen R
IgG: Immunoglobulin G
IHC: Immunohistochemistry
m6A: N6-methyladenosine
MeRIP: Methylated RNA immunoprecipitation
METTL14: Methyltransferase-like 14
METTL3: Methyltransferase-like 3
OS: Overall survival
RIP: RNA immunoprecipitation
RNA-seq: RNA-sequencing
RT-qPCR: Reverse transcription quantitative real-time PCR
SAM: S-adenosylmethionine
TCGA: The Cancer Genome Atlas
TMA: Tissue microarrays
WTAP: Wilms tumor 1-associated protein
