# Supplementary material for: WTAP facilitates progression of hepatocellular carcinoma via m6A-HuR-dependent epigenetic silencing of ETS1
Source: Mol Cancer. 2019 Aug 22;18:127. doi: 10.1186/s12943-019-1053-8 (PMC6704583; doi:10.1186/s12943-019-1053-8)
Supplement: Supplementary file 5 — Data S1. Potential m6A sites of ETS1 and related primers in this work. (DOCX 16 kb) [file 12943_2019_1053_MOESM5_ESM.docx]

**Data S1. Potential m6A sites of ETS1 and related primers**

Potential m6A sites of ETS1 were shown as follows based on the prediction via RMBase v2.0 and SRAMP. According to supporting numbers (supportNum) in RMBase v2.0, adenines were labeled as different colors. To be specific, red and blue represented more than 30 (highly supported) and 15 to 30 (moderately supported), respectively. Besides, scores provided by SRAMP included “very high” or “high”, which were highlighted with yellow or green, respectively. And underlined sequences indicated the forward or reverse primers.

**>ETS1-3’UTR**

TGGCACTGAAGGGGCTGGGGAA**A**CCCTGCTGAGACCTTCCAAGG**A**CAGCCGTGTTGGTTGG**A**CTCTGAATTTTGAATTGTTATTCTATTTTTTATTTTCCAGAACTCATTTTTTACCTTCAGGGGTGGGAGCTAAGTCAGTTGCAGCTGTAATCAATTGTGCGCAGTTGGGAAAGGAAAGCCAGGACTTGTGGGGTGGGTGGGACCAGAAATTCTTGAGCAAATTTTCAGGAGAGGGAGAAGGGCCTTCTCAGAAGCTTGAAGGCTCTGGCTTAACAGAGAAAGAGACTAATGTGTCCAATCATTTTTAAAAATCATCCATGAAAAAGTGTCTTGAGTTGTGGACCCATTAGCAAGTGACATTGTCACATCAGAACTCATGAAACTGATGTAAGGCAATTAATTTGCTTCTGTTTTTAGGTCTGGGAGGGCAAAAAAGAGGTGGGTGGGATGAAACATGTTTTGGGGGGGGATGCACTGAAAATCTGAGAACTATTTACCTATCACTCTAGTTTTGAAGCAAAGATGGACTTCAGTGGGGAGGGGCCAAAACCGTTGTTGTGTTAAAATTTATTTTATTAAATTTTGTGCCAGTA

**>ETS1-5’UTR**

GAGAGCGAACGAGGCAGCCGCCGGCGGAGAGGAGGGAGCGGGCGAGGGCCGGGCAGGAGGAGCGGGCGCGGCGCGGGCGAGGCTGGGACCCGAGCGCGCTCACTTCGCCGCAAAGTGCCAACTTCCCCTGGAGTGCCGGGCGCGCACCGTCCGGGCGCGGGGGAAAGAAAGGCAGCGGGAATTTGAGATTTTTGGGAAGAAAGTCGGATTTCCCCCGTCCCCTTCCCCCTGTTACTAATCCTCATTAAAAAGAAAAACAACAGTAACTGCAAACTTGCTACCATCCCGTACGTCCCCCACTCCTGGCACC

**>ETS1-CDS**

ATGAAGGCGGCCGTCGATCTCAAGCCGACTCTCACCATCATCAAGACGGAAAAAGTCGATCTGGAGCTTTTCCCCTCCCCGGATATGGAATGTGCAGATGTCCCACTATTAACTCCAAGCAGCAAAGAAATGATGTCTCAAGCATTAAAAGCTACTTTCAGTGGTTTCACTAAAGAACAGCAACGACTGGGGATCCCAAAAGACCCCCGGCAGTGGACAGAAACCCATGTTCGGGACTGGGTGATGTGGGCTGTGAATGAATTCAGCCTGAAAGGTGTAGACTTCCAGAAGTTCTGTATGAATGGAGCAGCCCTCTGCGCCCTGGGTAAAGACTGCTTTCTCGAGCTGGCCCCAGACTTTGTTGGGGACATCTTATGGGAACATCTAGAGATCCTGCAGAAAGAGGATGTGAAACCATATCAAGTTAATGGAGTCAACCCAGCCTATCCAGAATCCCGCTATACCTCGGATTACTTCATTAGCTATGGTATTGAGCATGCCCAGTGTGTTCCACCATCGGAGTTCTCAGAGCCCAGCTTCATCACAGAGTCCTATCAGACGCTCCATCCCATCAGCTCGGAAGAGCTCCTCTCCCTCAAGTATGAGAATGACTACCCCTCGGTCATTCTCCGAGACCCTCTCCAGACAGACACCTTGCAGAATGACTACTTTGCTATCAAACAAGAAGTCGTCACCCCAGACAACATGTGCATGGGGAGGACCAGTCGTGGTAAACTCGGGGGCCAGGACTCTTTTGAAAGCATAGAGAGCTACGATAGTTGTGATCGCCTCACCCAGTCCTGGAGCAGCCAGTCATCTTTCAACAGCCTGCAGCGTGTTCCCTCCTATGACAGCTTCGACTCAGAGGACTATCCGGCTGCCCTGCCCAACCACAAGCCCAAGGGCACCTTCAAGGACTATGTGCGGGACCGTGCTGACCTCAATAAGGACAAGCCTGTCATTCCTGCTGCTGCCCTAGCTGGCTACACAGGCAGTGGACCAATCCAGCTATGGCAGTTTCTTCTGGAATTACTCACTGATAAATCCTGTCAGTCTTTTATCAGCTGGACAGGAGATGGCTGGGAATTCAAACTTTCTGACCCAGATGAGGTGGCCAGGAGATGGGGAAAGAGGAAAAACAAACCTAAGATGAATTATGAGAAACTGAGCCGTGGCCTACGCTACTATTACGACAAAAACATCATCCACAAGACAGCGGGGAAACGCTACGTGTACCGCTTTGTGTGTGACCTGCAGAGCCTGCTGGGGTACACCCCTGAGGAGCTGCACGCCATGCTGGACGTCAAGCCAGATGCCGACGAGTGA
