# Supplementary material for: WTAP facilitates progression of hepatocellular carcinoma via m6A-HuR-dependent epigenetic silencing of ETS1
Source: Mol Cancer. 2019 Aug 22;18:127. doi: 10.1186/s12943-019-1053-8 (PMC6704583; doi:10.1186/s12943-019-1053-8)
Supplement: Supplementary file 6 — Data S2. Sequences of wild-type or mutant reporter plasmids in this work. (DOCX 15 kb) [file 12943_2019_1053_MOESM6_ESM.docx]

**Data S2. Sequences of cDNA fused in Luciferase reporters**

Wild-type and mutant cDNA of ETS1 fused in luciferase reporter were shown as follows (underlined bases in red represented non-mutated or mutated sites; highlighted bases represented the beginning of 3’ UTR).

Wild-type cDNA：

ATTATGAGAA**A**CTGAGCCGTGGCCTACGCTACTATTACGACAAAA**A**CATCATCCACAAG**A**CAGCGGGGAAACGCTACGTGTACCGCTTTGTGTGTGACCTGCAGAGCCTGCTGGGGTACACCCCTGAGGAGCTGCACGCCATGCTGGACGTCAAGCCAGATGCCGACGAGTGATGGCACTGAAGGGGCTGGGGAA**A**CCCTGCTGAG**A**CCTTCCAAGG**A**CAGCCGTGTTGGTTGG**A**CTCTGAATTTTGAATTGTTATTCTATTTTTTATTTTCCAGA**A**CTCATTTTTTACCTTCAGGGGTGGGAGCTAAGTCAGTTGCAGCTGTAATCAATTGTGCGCAGTTGGGAAAGGAAAGCCAGG**A**CTTGTGGGGTGGGTGGG**A**CCAGAAATTCTTGAGCAAATTTTCAGGAGAGGGAGAAGGGCCTTCTCAGAAGCTTGAAGGCTCTGGCTTAACAGAGAAAGAG**A**CTAATGTGTCCAATCATTTTTAAAAATCATCCATGAAAAAGTGTCTTGAGTTGTGGACCCATTAGCAAGTGACATTGTCACATCAGAACTCATGAAACTGATGTAAGGCAATTAATTTGCTTCTGTTTTTAGGTCTGGGAGGGCAAAAAAGAGGTGGGTGGGATGAAACATGTTTTGGGGGGGGATGCACTGAAAATCTGAGAACTATTTACCTATCACTCTAGTTTTGAAGCAAAGATGGACTTCAGTGGGGAGGGGCCAAAACCGTTGTTGTGTTAAAATTTATTTTATTAAATTTTGTGCCAGTA

Mutant 1 (11 mutated points) cDNA：

ATTATGAGAA**C**CTGAGCCGTGGCCTACGCTACTATTACGACAAAA**C**CATCATCCACAAG**C**CAGCGGGGAAACGCTACGTGTACCGCTTTGTGTGTGACCTGCAGAGCCTGCTGGGGTACACCCCTGAGGAGCTGCACGCCATGCTGGACGTCAAGCCAGATGCCGACGAGTGATGGCACTGAAGGGGCTGGGGAA**C**CCCTGCTGAG**C**CCTTCCAAGG**C**CAGCCGTGTTGGTTGG**C**CTCTGAATTTTGAATTGTTATTCTATTTTTTATTTTCCAGA**C**CTCATTTTTTACCTTCAGGGGTGGGAGCTAAGTCAGTTGCAGCTGTAATCAATTGTGCGCAGTTGGGAAAGGAAAGCCAGG**C**CTTGTGGGGTGGGTGGG**C**CCAGAAATTCTTGAGCAAATTTTCAGGAGAGGGAGAAGGGCCTTCTCAGAAGCTTGAAGGCTCTGGCTTAACAGAGAAAGAG**C**CTAATGTGTCCAATCATTTTTAAAAATCATCCATGAAAAAGTGTCTTGAGTTGTGGACCCATTAGCAAGTGACATTGTCACATCAGAACTCATGAAACTGATGTAAGGCAATTAATTTGCTTCTGTTTTTAGGTCTGGGAGGGCAAAAAAGAGGTGGGTGGGATGAAACATGTTTTGGGGGGGGATGCACTGAAAATCTGAGAACTATTTACCTATCACTCTAGTTTTGAAGCAAAGATGGACTTCAGTGGGGAGGGGCCAAAACCGTTGTTGTGTTAAAATTTATTTTATTAAATTTTGTGCCAGTA

Mutant 2 (4 mutated points) cDNA:

ATTATGAGAAACTGAGCCGTGGCCTACGCTACTATTACGACAAAAACATCATCCACAAGACAGCGGGGAAACGCTACGTGTACCGCTTTGTGTGTGACCTGCAGAGCCTGCTGGGGTACACCCCTGAGGAGCTGCACGCCATGCTGGACGTCAAGCCAGATGCCGACGAGTGATGGCACTGAAGGGGCTGGGGAA**C**CCCTGCTGAG**C**CCTTCCAAGG**C**CAGCCGTGTTGGTTGG**C**CTCTGAATTTTGAATTGTTATTCTATTTTTTATTTTCCAGAACTCATTTTTTACCTTCAGGGGTGGGAGCTAAGTCAGTTGCAGCTGTAATCAATTGTGCGCAGTTGGGAAAGGAAAGCCAGGACTTGTGGGGTGGGTGGGACCAGAAATTCTTGAGCAAATTTTCAGGAGAGGGAGAAGGGCCTTCTCAGAAGCTTGAAGGCTCTGGCTTAACAGAGAAAGAGACTAATGTGTCCAATCATTTTTAAAAATCATCCATGAAAAAGTGTCTTGAGTTGTGGACCCATTAGCAAGTGACATTGTCACATCAGAACTCATGAAACTGATGTAAGGCAATTAATTTGCTTCTGTTTTTAGGTCTGGGAGGGCAAAAAAGAGGTGGGTGGGATGAAACATGTTTTGGGGGGGGATGCACTGAAAATCTGAGAACTATTTACCTATCACTCTAGTTTTGAAGCAAAGATGGACTTCAGTGGGGAGGGGCCAAAACCGTTGTTGTGTTAAAATTTATTTTATTAAATTTTGTGCCAGTA
