# Supplementary figures and images for: WTAP facilitates progression of hepatocellular carcinoma via m6A-HuR-dependent epigenetic silencing of ETS1
Source: Mol Cancer. 2019 Aug 22;18:127. doi: 10.1186/s12943-019-1053-8 (PMC6704583; doi:10.1186/s12943-019-1053-8)

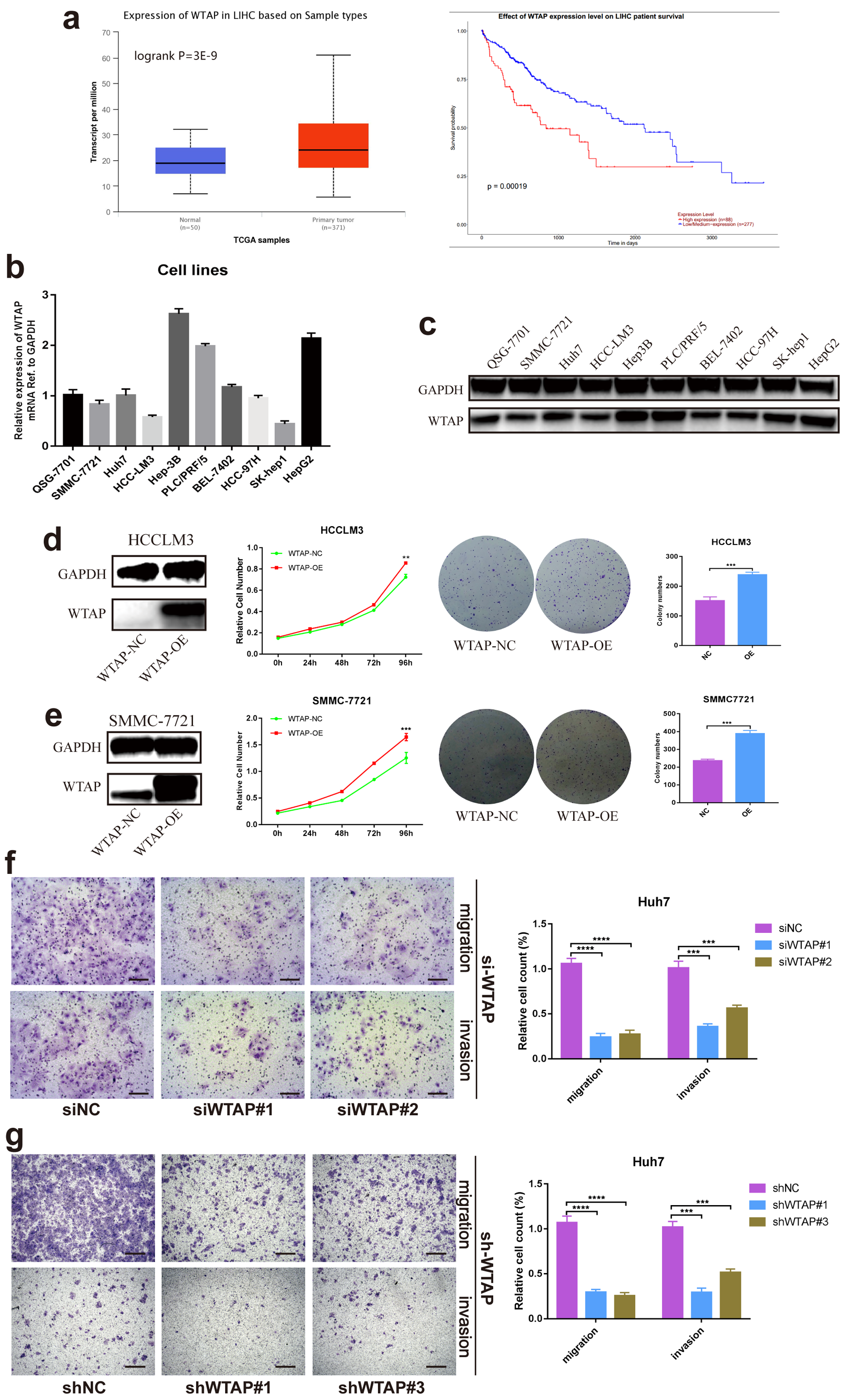

Supplement: Supplementary file 7 — Figure S1. Expression of WTAP in cell lines and functional investigations of WTAP. a Expression and survival analysis of WTAP in HCC (data from TCGA, analyzed with UALACN, http://ualcan.path.uab.edu/analysis.html); b, c mRNA (b) and protein (c) level of WTAP in an immortalized hepatic cell line (QSG-7701) and nine HCC cell lines; d, e Negative control vector or Flag-WTAP was transfected into HCCLM3 (d) or SMMC-7721 (e) with the overexpression efficiency determined. Proliferation capacities were detected by CCK-8, colony formation assay; f, g Representative images and bar charts of cell migration and invasion ability in Huh7 cells with WTAP knockdown (siRNA or shRNA) or negative control detected by transwell and matrigel transwell assays (scale bar, 100 μm). (TIF 3566 kb) [file 12943_2019_1053_MOESM7_ESM.tif]

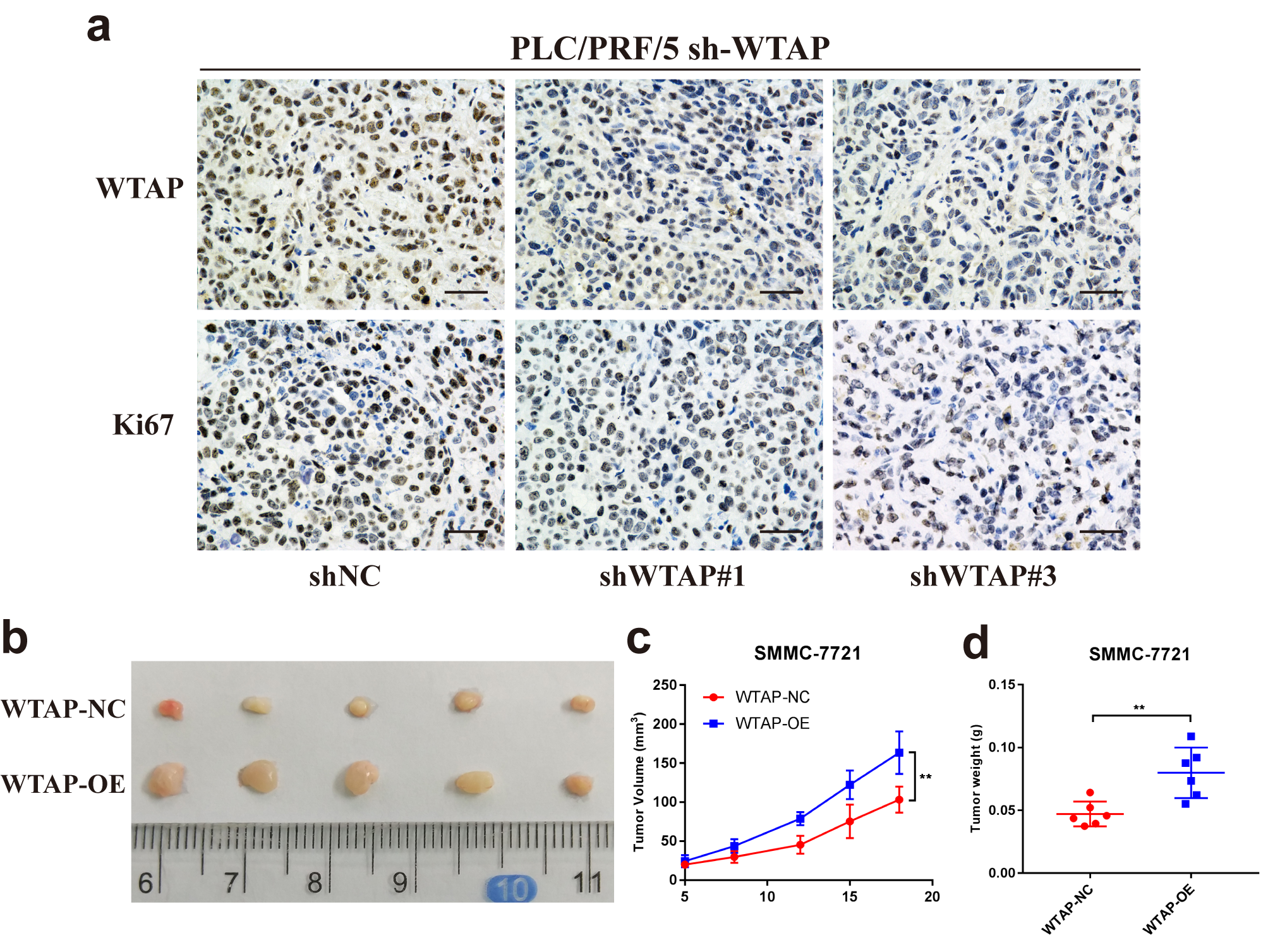

Supplement: Supplementary file 8 — Figure S2. The impact of WTAP in vivo. a The level of WTAP and Ki67 in xenograft tumor tissues was detected by IHC (scale bar, 50 μm; magnification, 400X); b-d Tumor growth curve (c) of SMMC7721 with stable WTAP epitopic expression cells in a xenograft mouse model was based on the tumor sizes. And the photography (b) and tumor weights (d) were recorded to exhibit the growth difference within the influence of WTAP. (TIF 4248 kb) [file 12943_2019_1053_MOESM8_ESM.tif]

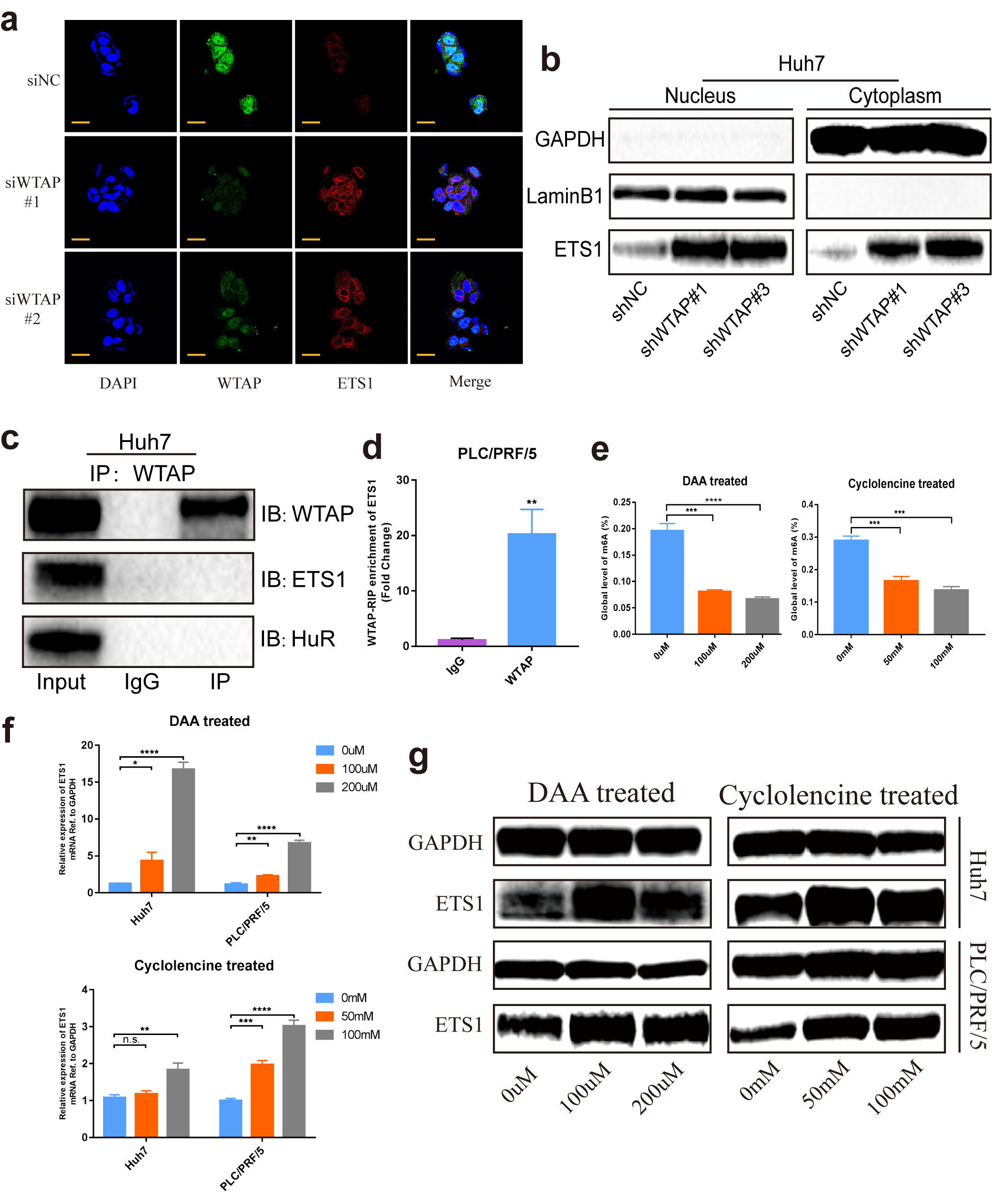

Supplement: Supplementary file 9 — Figure S3. Mechanisms of WTAP-mediated modulation on ETS1. a Representative immunofluorescence images of Huh7 cells with the deficiency of WTAP to determine the subcellular distribution and expression of WTAP and ETS1 (scale bar, 30 μm). WTAP mainly localized in nucleus while ETS1 mainly in cytoplasm. However, fluorescence intensity of ETS1 significantly augmented in either cytosolic or nuclear regions (especially in nuclear membrane); b Cytosolic and nuclear separation analysis was conducted to examine the expression of ETS1 within subcellular components under WTAP silencing; c The protein interaction between WTAP and ETS1 or HuR was precluded by Co-IP assay; d WTAP-RIP was applied to verify the enrichment of ETS1 mRNA by WTAP antibody; e Overall level of m6A was determined by RNA methylation quantification assay after the treatment of DAA and cyclolencine in Huh7 cell with diverse concentration, respectively; f and g Huh7 and PLC/PRF/5 was treated with DAA in the concentration of 0uM, 100uM, 200uM; Another panel, was treated with cyclolencine in the concentration of 0 mM, 50 mM, 100 mM. And the expression of ETS1 was detected in RNA (f) and protein (g) level. (TIF 1651 kb) [file 12943_2019_1053_MOESM9_ESM.tif]

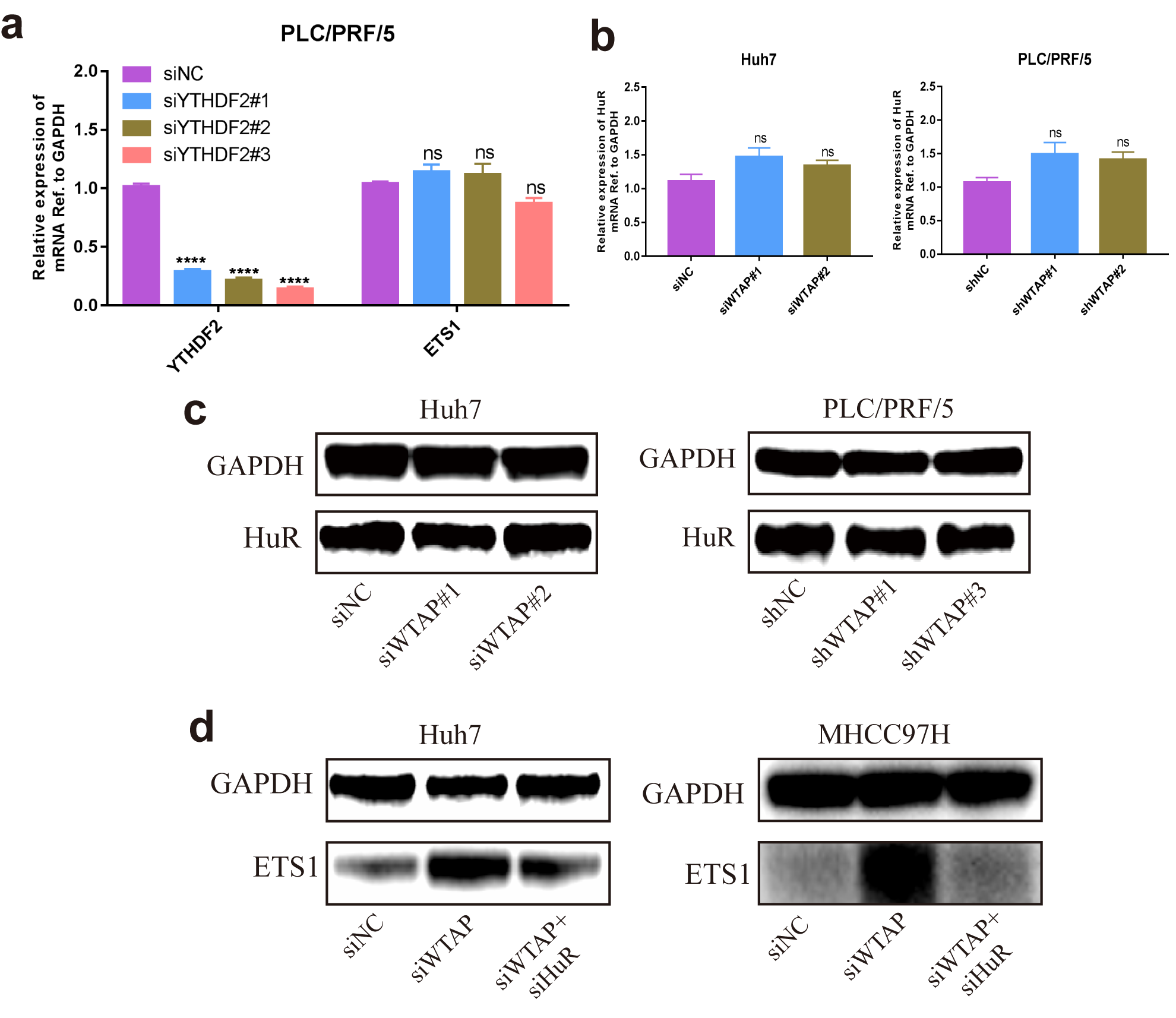

Supplement: Supplementary file 10 — Figure S4. Mechanisms of HuR-involved regulation of ETS1. a YTHDF2 was knockdown in PLC/PRF/5 without any variation in ETS1 expression; b and c WTAP was knockdown followed by qRT-PCR (b) and western blotting (c) to estimate the alteration of HuR; d WTAP-inactivation caused a striking enlargement of ETS1, which could be rescued by knockdown of HuR. (TIF 755 kb) [file 12943_2019_1053_MOESM10_ESM.tif]

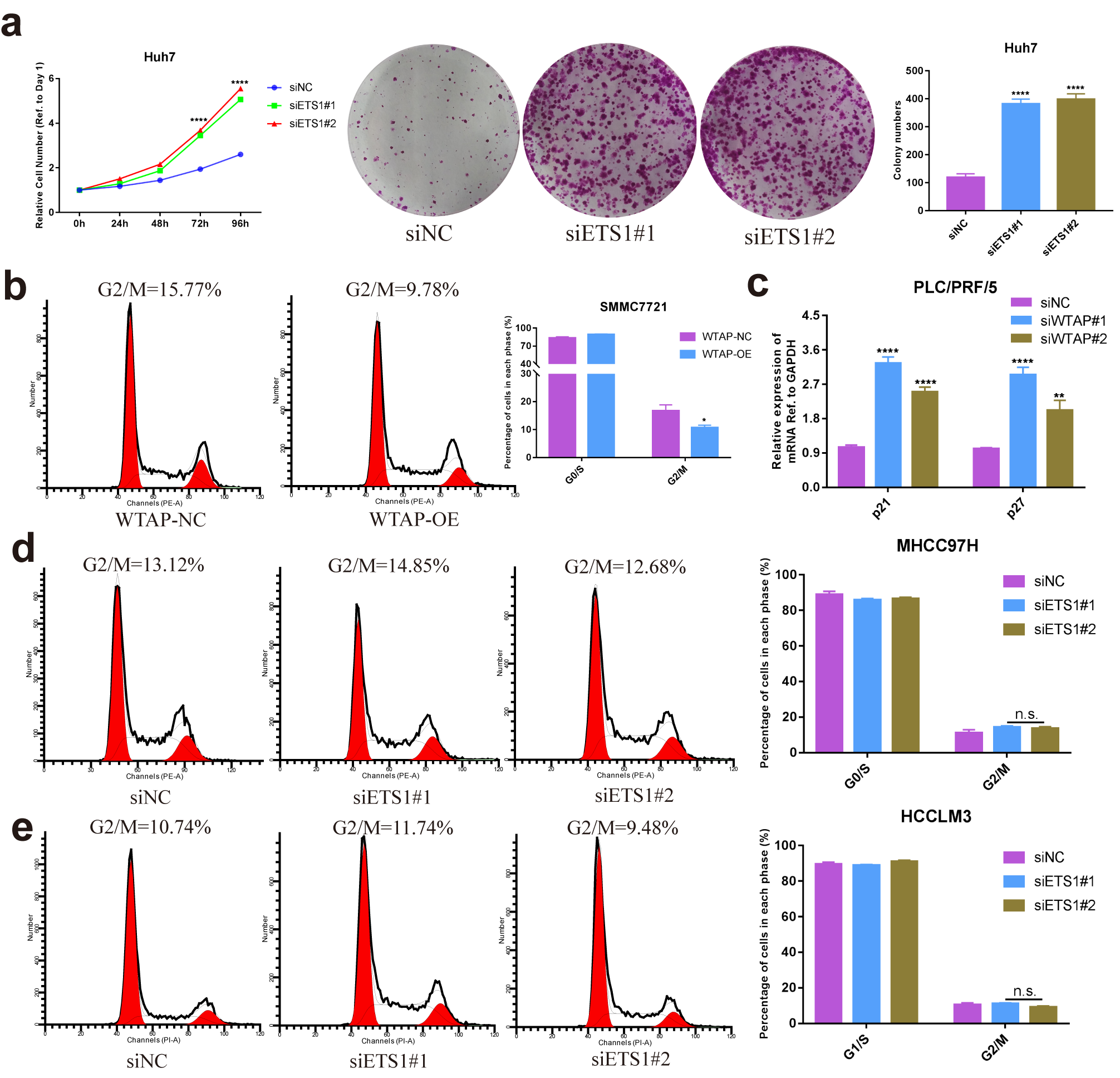

Supplement: Supplementary file 11 — Figure S5. Proliferation and cell cycle investigations of ETS1. a CCK8 and colony formation assay were performed to test propagation ability of Huh7 cell where ETS1 was knockdown; b Cell cycle distribution was analyzed by flow cytometry in SMMC-7721 cell where WTAP was overexpression, with bar charts indicating the percentage of cells in each phase; c RT-qPCR was used to find changes of p21 and p27 when WTAP was knockdown in PLC/PRF/5; d and e Flow cytometric analysis was conducted in MHCC97H (d) and HCCLM3 (e) cell with the inactivation of ETS1. (TIF 1434 kb) [file 12943_2019_1053_MOESM11_ESM.tif]

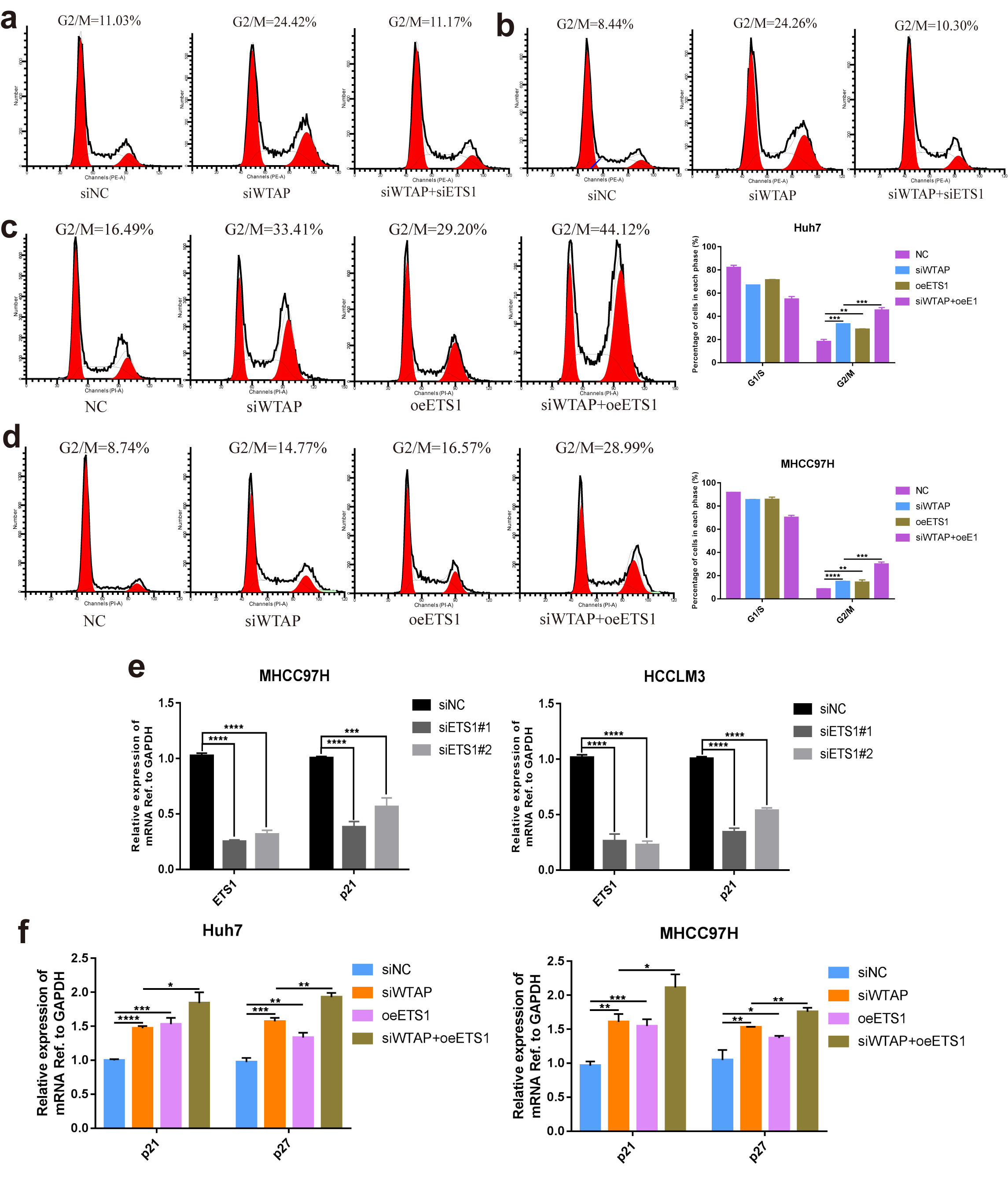

Supplement: Supplementary file 12 — Figure S6. Cell cycle inquiry in WTAP/ETS1 rescued cells a and b Rescue assays of cell cycle distribution were performed in WTAP-silenced MHCC97H (a) and HCCLM3 (b) cells with or without siETS1; c and d Cell cycle distribution were performed in WTAP-silenced Huh7 (c) and MHCC97H (d) cells with or without ETS1 overexpression; e Expression of p21 or p27 was measured with the reduction of ETS1 in RNA level; f Expression of p21 and p27 was measured in WTAP-knockdown Huh7 and MHCC97H cells with or without ETS1 overexpression. (TIF 1000 kb) [file 12943_2019_1053_MOESM12_ESM.tif]

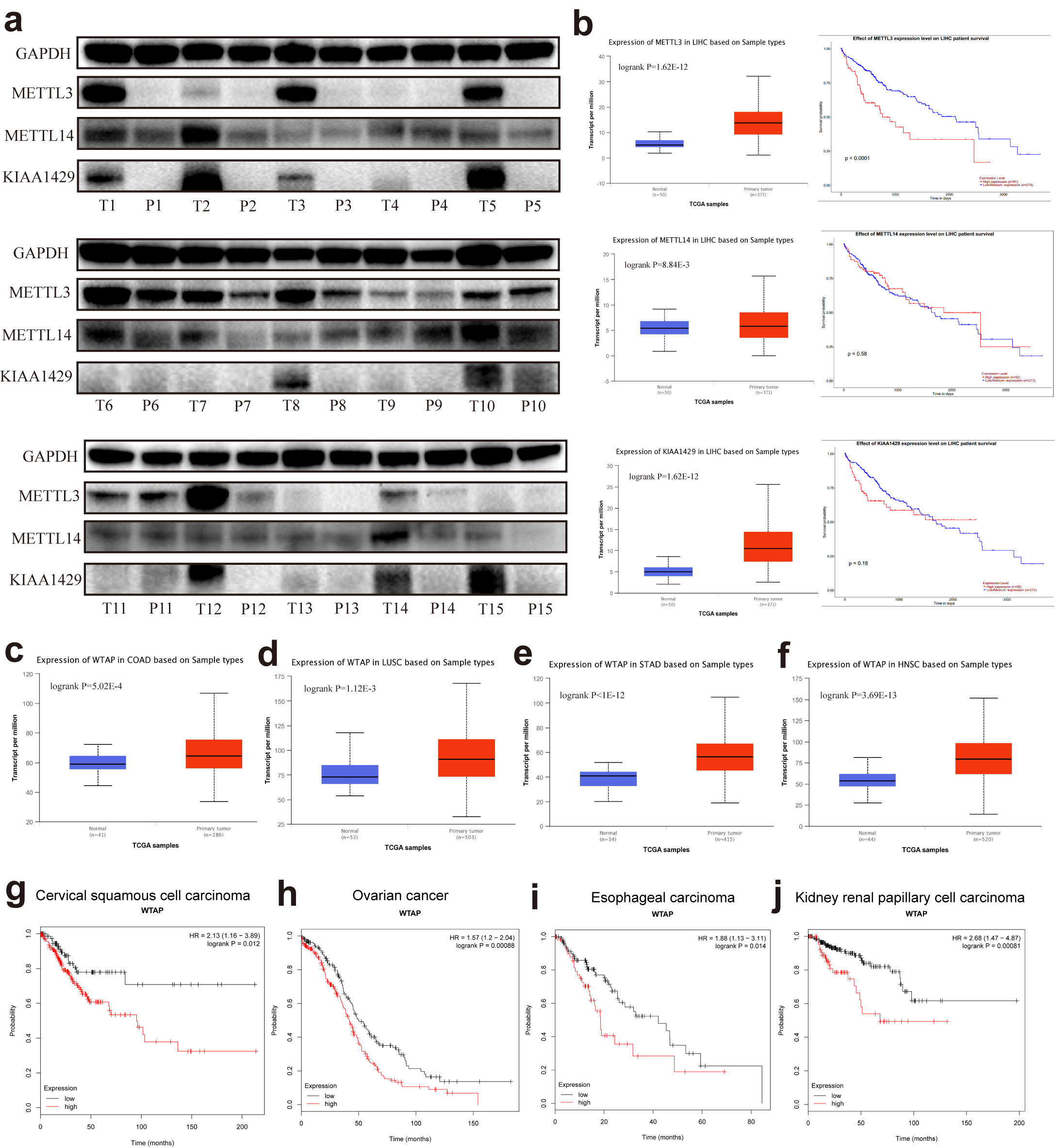

Supplement: Supplementary file 13 — Figure S7. Pan-cancer expression and survival analysis of WTAP and other m6A-related enzymes. a Expression of METTL3, METT14 and KIAA1429 protein was analyzed by western blotting in 15 pairs of HCC tissues; (T: tumor; P: peritumor); b Expression and survival analysis of three enzymes mentioned above (data from TCGA, analyzed with UALACN); c-f Expression of WTAP in tumor and para-tumor tissues of Colon adenocarcinoma (c), Lung squamous cell carcinoma (d), Stomach adenocarcinoma (e) and Head and Neck squamous cell carcinoma (f) (data from TCGA, analyzed with UALACN); g-j Overall survival curves of HCC patients according to the expression of WTAP in four tumors (data from TCGA, analyzed with KM plotter, http://kmplot.com/analysis/). (TIF 1805 kb) [file 12943_2019_1053_MOESM13_ESM.tif]

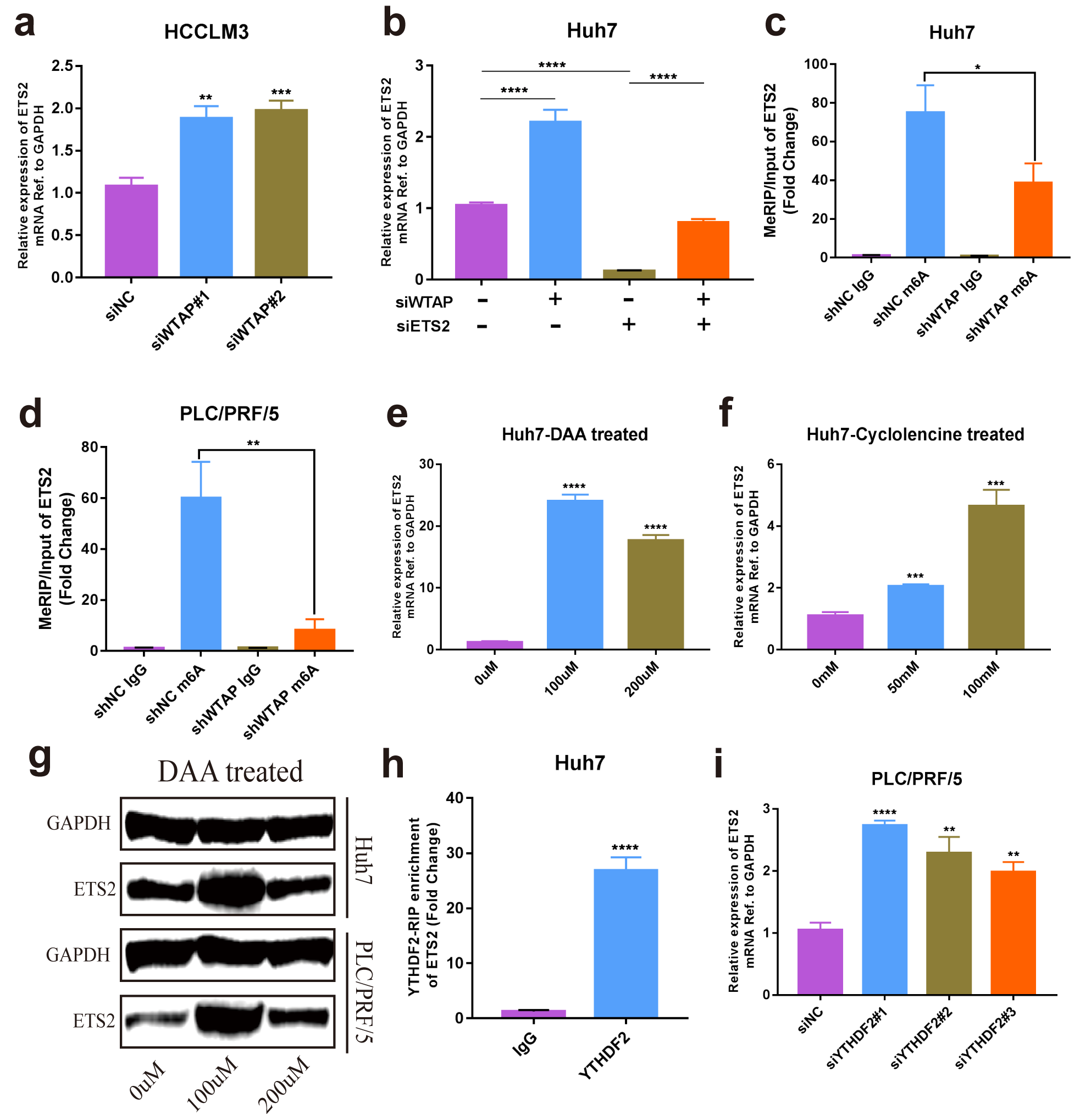

Supplement: Supplementary file 14 — Figure S8. ETS2 was negatively regulated by WTAP via m6A-YTHDF2 pathway. a Expression of ETS2 was measured following silencing of WTAP in HCCLM3 cell; b A retrieval assay was performed with the knockdown of WTAP or ETS2; c, d WTAP-mediated m6A modification of ETS2 was assessed by MeRIP-qPCR with specific primers; e, f Expression of ETS2 after treatment of DAA (e) and cyclolencine (f) by RT-qPCR; g Expression of ETS2 after treatment of DAA was detected by western blotting; h YTHDF2-RIP was applied to evaluate the enrichment of ETS2 by YTHDF2 antibody; i Expression of ETS2 was surveyed upon the knockdown of YTHDF2. (TIF 827 kb) [file 12943_2019_1053_MOESM14_ESM.tif]

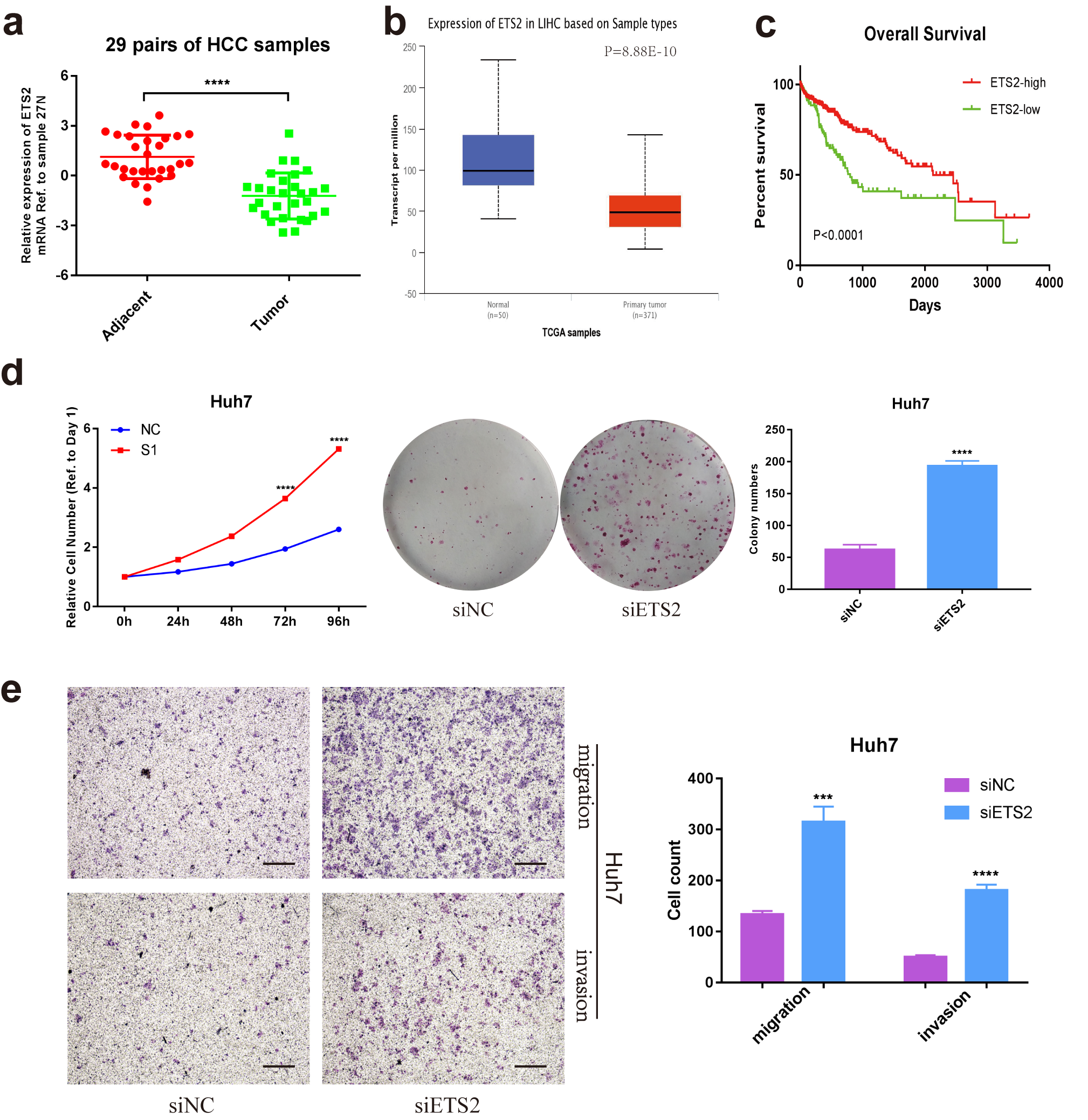

Supplement: Supplementary file 15 — Figure S9. Expression, survival and functional analyses of ETS2. a Expression of ETS2 in HCC cancerous and normal tissues from 29 pairs of HCC samples; b Expression of ETS2 in HCC cancerous and normal tissues based on TCGA datasets; c Overall survival of HCC patients grouped by the level of ETS2; d CCK-8 and colony formation assays was applied to determine the viability of ETS2 knockdown cell; e Transwell assays was utilized to evaluate the motility of ETS2 knockdown cells (scale bar, 100 μm). (TIF 3161 kb) [file 12943_2019_1053_MOESM15_ESM.tif]
